# Real‐Time Monitoring of Atherosclerotic Plaque Using β‐Galactosidase‐Activated Photoacoustic Tomography

**DOI:** 10.1002/smll.202506731

**Published:** 2025-09-12

**Authors:** Yifan Zhou, Hang Yang, Handi Deng, Feng Zhang, Junjie Jiang, Sibo Yang, Xiaodi Sun, Jiehong Wu, Yanan Li, Huijuan Jin, Hao Wang, Cheng Ma, Li‐Li Li, Bo Hu

**Affiliations:** ^1^ Department of Neurology, Union Hospital, Tongji Medical College Huazhong University of Science and Technology Wuhan 430022 P. R. China; ^2^ Department of Electronic Engineering, Beijing National Research Center for Information Science and Technology Tsinghua University Beijing 100084 P. R. China; ^3^ Institute for Intelligent Healthcare Tsinghua University Beijing 100084 P. R. China; ^4^ CAS Center for Excellence in Nanoscience, CAS Key Laboratory for Biological Effects of Nanomaterials and Nanosafety National Center for Nanoscience and Technology (NCNST) No. 11 Beiyitiao, Zhongguancun Beijing 100190 P. R. China; ^5^ IDG/McGovern Institute for Brain Research at Tsinghua University Beijing 100084 P. R. China; ^6^ School of Material Science and Engineering Beijing Institute of Technology Beijing 100081 P. R. China

**Keywords:** atherosclerosis, bioactivated in vivo assembly, nanoprobe, photoacoustic tomography imaging, senescence‐associated β‐galactosidase

## Abstract

The early monitoring and risk assessment of atherosclerotic progression is crucial yet challenging due to its intricate and dynamic pathological process. A lipo‐nanoprobe incorporating chlorophyll derivatives is developed, which enables enzyme‐triggered in situ J‐aggregation‐induced red‐shift, allowing detection at 710 nm with a linear relationship to senescence‐associated β‐galactosidase (SA‐β‐gal), an essential marker of atherosclerotic formation and progression. The photoacoustic histological analysis conducted using the nanoprobe confirmed the accurate visualization of plaque microenvironment SA‐β‐gal activity, enabling semi‐quantitation of plaque progression at different time points. By employing dynamic multi‐wavelength imaging, the nanoprobe presented a specific SA‐β‐gal‐dependent absorption at 710 nm in vivo. Utilizing photoacoustic tomography imaging, 3D images of plaques were reconstructed and quantitative analysis was performed on the extent and distribution of senescence in the total aortic vascular wall. The monitored signal revealed significant trends in relation to cellular senescence phenotype, particularly in vascular smooth muscle cells (VSMCs), which are crucial for clinically assessing the impact of cellular processes that contribute to plaque formation and expansion during the progression of atherosclerosis. This study demonstrates the utility of SA‐β‐gal‐responsive photoacoustic imaging for detecting plaque pathological progression and highlights its advantage for early monitor plaque formation in individuals at risk of developing atherosclerosis.

## Graphical abstract

Detecting Vascular Senescence: Novel Enzyme‐Responsive Photoacoustic Nanoprobes for Atherosclerotic Plaque Imaging. A lipid nanoprobe is developed that detects senescence‐associated β‐galactosidase in plaques via enzyme‐triggered spectral shifts. This platform enables dynamic tracking of atherosclerotic progression, 3D visualization on the extent and distribution of senescence in the total vascular wall, and quantitative algorithms of senescence analysis—a powerful tool for early atherosclerosis risk assessment.

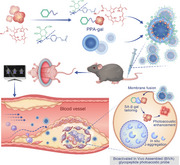

## 1 Introduction

Atherosclerosis represents a leading cause of acute thrombotic events and subsequent stroke or cardiovascular diseases,^[^ ^1^ ^]^ and affects more than 70% of individuals aged 65 and older globally. Beginning with the accumulation of excess low‐density lipoprotein (LDL) cholesterol in the subendothelial space, atherosclerosis is characterized by a complex and dynamic pathological process that includes activation of macrophages and smooth muscle cells (VSMCs), with progressive inflammation.^[^ ^2^ ^]^ The first line of medical treatment for atherosclerosis is with lipid‐lowering agents to reduce plasma LDL‐c.^[^ ^3^ ^]^ While LDL‐c detection offers limited insight into the atherosclerotic process, more than 50% of patients undergoing treatment fail to experience the therapeutic benefits.^[^ ^4^, ^5^ ^]^ How to make an applicable decision on the corresponding diagnosis and treatment based on underlying pathological processes remains to be further explored.

At present, clinical monitoring of atherosclerosis is highly dependent on the morphological features of vascular wall.^[^ ^6^, ^7^ ^]^ Although both invasive strategies (e.g., optical coherence tomography) and non‐invasive imaging approaches (e.g., ultrasound or magnetic resonance imaging) have been tested for identifying plaque components, morphological features, and plaque vulnerability,^[^ ^6^ ^]^ the progression of plaque is difficult to detect in early stages and can accelerate, leading to rapid thrombus formation, if undetected prior to reaching certain thresholds of pathological severity.^[^ ^8^, ^9^ ^]^

In recent years, it has become widely accepted that accurate diagnostic and therapeutic strategies require a comprehensive understanding of both plaque composition as well as the pathophysiology of atherosclerosis.^[^ ^10^, ^11^ ^]^ To this end, several previous efforts have focused on developing molecular imaging approaches targeting the cellular and molecular components of vulnerable atherosclerotic plaques.^[^ ^10^ ^]^ However, plaque‐targeted pathological imaging still faces some crucial limitations and challenges, such as toxicity and/or severe side effects of the nanoscale imaging agent that damage endothelial cells (ECs).^[^ ^12^ ^]^ Moreover, the low sensitivity of some imaging modalities restricts current contrast agents to targeting a few highly expressed molecules within atherosclerotic plaques, but which vary in different stages of atherosclerosis progression.^[^ ^6^, ^13^ ^]^ Vascular senescence begins early in the pathogenesis of atherosclerosis, becoming more widespread throughout its pathological progression.^[^ ^14^ ^]^ Senescence markers used to track this development include elevated senescence‐associated β‐galactosidase (SA‐β‐gal) activity, and p16^Ink4a^, p53, and p21 expression.^[^ ^15^, ^16^ ^]^ The former of these, SA‐β‐gal, is an active marker of cell senescence, which can be observed early in the plaque formation process. SA‐β‐gal positive foam cells secrete key atherogenic chemokines and induce inflammatory cascades, which further promote rapid plaque expansion and instability.^[^ ^17^ ^]^ Senescence of smooth muscle cells (VSMCs) accelerates lipid core enlargement and fibrous cap rupture.^[^ ^17^, ^18^, ^19^, ^20^ ^]^ Thus, some studies indicate that senolytic drugs and genetic manipulations reduce atherogenesis.^[^ ^14^, ^21^ ^]^ Still, there are still some studies that show either no effect or detrimental changes that could promote plaque rupture.^[^ ^22^ ^]^ Overall, pathological processes specifically associated with senescent cells may serve as effective targets in both diagnostic imaging and therapeutic strategies for atherosclerosis.

Unfortunately, currently available methods for monitoring atherosclerotic plaques in clinics lack sufficient sensitivity to detect nascent plaque formation.^[^ ^23^ ^]^ Non‐destructive photoacoustic imaging (PAI), which combines photoexcitation with ultrasound to provide deep tissue penetration and high spatial resolution, has shown considerable potential for clinical application.^[^ ^24^, ^25^ ^]^ Photoacoustic probes facilitate the conversion of laser energy into thermoelastic expansion, the signal of which is recorded by an ultrasonic detector and then is reconstructed into image data.^[^ ^25^, ^26^ ^]^ Therefore, we hypothesized that a specific biochemical event, i.e., SA‐β‐gal expression, which contributes to plaque formation and expansion, could be detected using a probe to enable earlier monitoring of atherosclerosis pathogenesis. Moreover, dynamic, non‐invasive monitoring of SA‐β‐gal expression and early plaque formation by high contrast and deep penetration PAI could facilitate detailed investigation of the various cellular processes driving plaque formation and expansion during atherosclerotic progression, especially SMC senescence.

**Figure 1:**
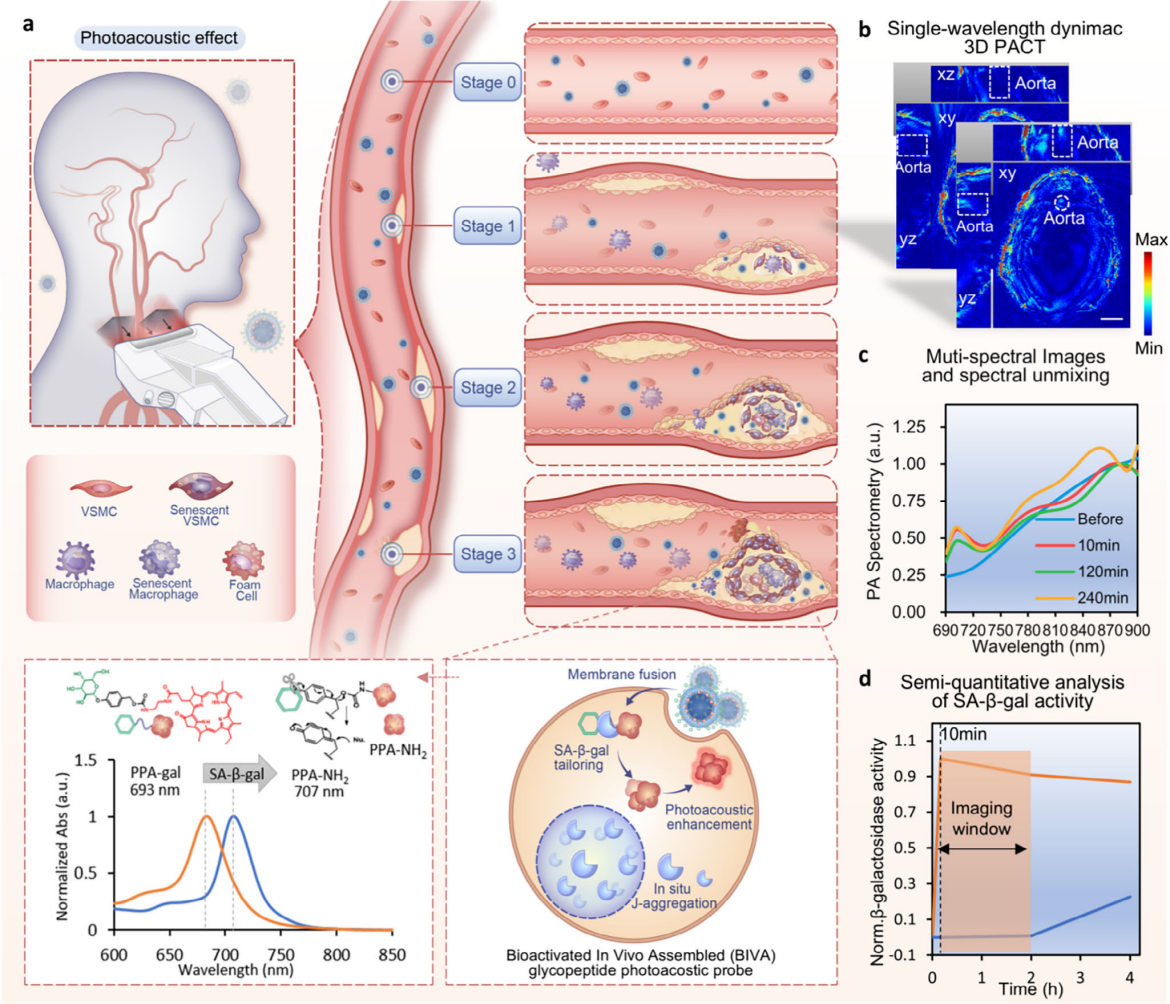
Schematic illustration of PPA‐galactose‐LNP for atherosclerosis detection. a) The study employs Pyropheophorbide‐a (PPA‐galactose), modified with the SA‐β‐galactosidase (SA‐β‐gal) responsible group, as a photoacoustic probe encapsulated within nanoliposomes for the purpose of in vivo photoacoustic imaging. Upon interaction with SA‐β‐gal in senescent cells, PPA‐galactose undergoes self‐assembly into polymeric structures, which induces a spectral red shift and augments the photoacoustic signal specifically at 710 nm. Through this approach, β‐galactosidase can be visualized in different stages of atherosclerotic plaques, offering a dynamic enhancement that provides a comprehensive view of plaque progression. b) 3D photoacoustic computed tomography (PACT) can achieve dynamic detection of aortic plaque in ApoE^−/−^ mice. c) The application of a multispectral unmixing algorithm facilitates enhanced precision in localizing the region of interest. d) This methodology permits a semi‐quantitative evaluation of senescent subsequent to the administration of a nanoprobe in imaging window form 10 min to 2 h post injection.

In this work, we have developed a non‐invasive monitoring strategy utilizing a bioactivated in vivo assembly (BIVA) technique for nanoprobes targeting SA‐β‐gal to specifically detect elevated senescence events in plaques that contribute to the formation and progression of atherosclerosis, raising the possibility of making clinical stages for human carotid atherosclerotic plaques (**Figure** **1**a). This nanoprobe, PPA‐gal‐LNP (pyropheophorbide‐a‐galactose ‐modified lipid nanoparticle), was constructed using the chlorophyll derivative, galactose (gal)‐modified pyropheophorbide‐a (PPA), as the signal‐generating moiety, which is delivered by a liposome vehicle. Turbulence in circulation is well‐known to form at sites of vascular stenosis, which can facilitate the deposition and adhesion of lipid nanoparticles (LNPs).^[^ ^20^ ^]^ Fluorescence imaging of tissue sections from atherosclerosis model mice showed membrane fusion with the liposome vesicles and efficient penetration of the 100 nm PPA‐gal‐LNPs into plaques, which enabled imaging of intracellular SA‐β‐gal. Overexpression of SA‐β‐gal in senescent cells triggered a shift to red emission spectra of the chlorophyll derivative moiety through in situ J‐aggregation, which was detectable at 710 nm range (Figure 1a). Continuous non‐invasive 3D photoacoustic tomography (PACT) at 710 nm (Figure 1b) remained stable over a 10 min to 2 h window, effectively allowing monitoring of trends in cellular senescence phenotype, which directly participate in plaque formation and expansion. Furthermore, multi‐wavelength (670–900 nm) photoacoustic imaging (PAI) enables semi‐quantitative assessment of enzyme activity in vivo using a spectral unmixing analysis method developed by our team (Figure 1c,d). Photoacoustic tomography imaging assisted by our nanoprobe revealed the extent and distribution of senescence, providing higher sensitivity than magnetic resonance enhanced imaging and intravascular optical coherence tomography (OCT), the current gold standard for clinical assessment. In addition to its diagnostic potential, this method could also facilitate the investigation of cellular processes associated with plaque formation and expansion in atherosclerosis. Our nanoprobe‐based in vivo PAI matched well with 3D reconstructed images compared to histological analysis, further demonstrating the enhanced precision of our strategy. The PPA‐gal‐LNPs developed in this study showed considerable promise for enhancing accuracy in real‐time photoacoustic tomography imaging of early pathogenesis of atherosclerotic plaques.

## 2 Results and Discussion

### 2.1 Association Between SA‐β‐Gal Expression and Atherosclerotic Pathological Classification

**Figure 2:**
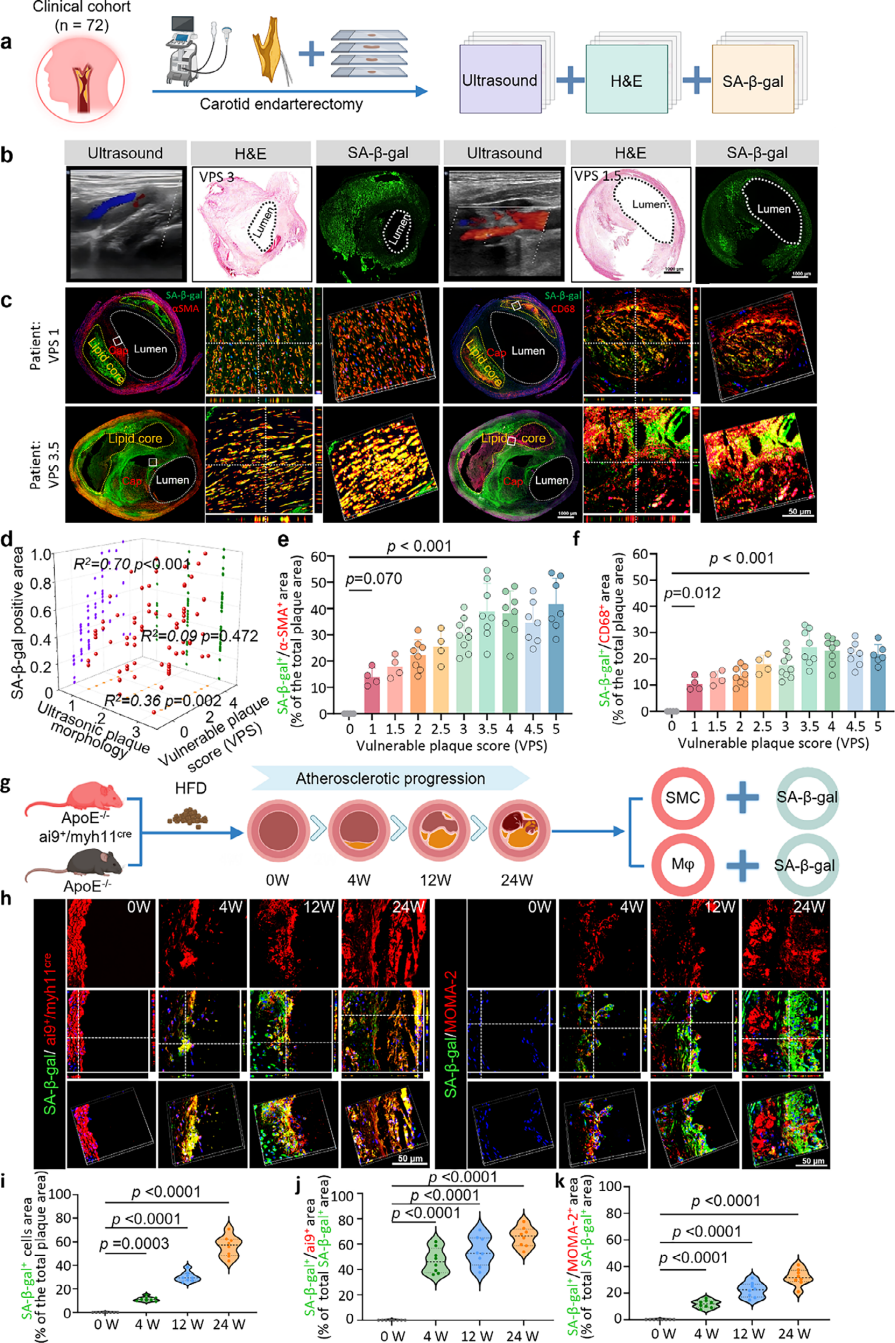
Plaque senescence is positively correlated with plaque progression in human plaques. a) Schematic for the comparison of SA‐β‐gal and vulnerable plaque score, ultrasonographic morphology of plaques in carotid artery plaques of patients undergoing CEA. b) Representative images of plaque hematoxylin and eosin (H&E) staining at various grading levels, alongside corresponding ultrasound imaging and senescence‐associated β‐galactosidase staining (SA‐β‐gal) staining (scale bar = 1 mm). c) Representative images of plaque immunofluorescence staining of VSMCs (red, α‐SMA), macrophages (red, CD68), and SA‐β‐gal (green) of plaque, SA‐β‐gal^+^ α‐SMA^+^ or SA‐β‐gal^+^ CD68^+^ double positive (yellow) (scale bar = 50 µm). d) Correlation analyses (red dots) examining relationships between SA‐β‐gal area, vulnerable plaque score, and ultrasound grade in patients with CEA. Yellow dots in the x‐y plane represent correlations between vulnerable plaque score and ultrasound grade. Purple dots in the x‐z plane represent correlations between vulnerable plaque score and SA‐β‐gal area. Green dots in the y‐z plane represent correlations between ultrasound grade and SA‐β‐gal area (*n* = 72). e‐f) Quantitative analysis of colocalized cells and vulnerable plaque scores. g) Schematic for Scheme for construction of different progression of atherosclerosis and immunofluorescence staining. h) Representative images of immunofluorescence staining of VSMCs (red), macrophages (red, MOMA‐2), and SA‐β‐gal (green) of plaque, SA‐β‐gal^+^ai9^+^ or SA‐β‐gal^+^MOMA‐2^+^ double positive (yellow) (scale bar = 50 µm). i) The proportion of SA‐β‐gal‐stained cells in different progression of atherosclerotic plaques (*n* = 8). j‐k) The proportion of SA‐β‐gal^+^ α‐SMA^+^ or SA‐β‐gal^+^ MOMA‐2^+^ double positive area in plaques of different degrees of progression (*n* = 8). Data in e‐f, i‐k are represented as mean ± s.d. Statistical significance was assessed via a one‐way ANOVA with post hoc Tukey's HSD test performed by GraphPad Prism. Data in d are compared by regression analysis performed by SPSS.

Atherosclerosis is driven by complex inflammatory and immune processes, creating a highly intricate biochemical environment and pathological condition. This complexity presents both challenges and opportunities for dynamic monitoring of the disease. Current molecular probes in atherosclerosis imaging can quantify vascular inflammation, early calcification, and plaque neoangiogenesis.^[^ ^7^ ^]^ However, these processes do not linearly correlate with plaque progression, as they tend to manifest at later stages of plaque development.^[^ ^10^, ^12^, ^13^ ^]^ The contribution of premature biological aging to atherosclerosis has become increasingly accepted among cardiac‐cerebral vascular disease researchers, largely due to evidence obtained by cell senescence markers.^[^ ^27^ ^]^ To investigate SA‐β‐gal expression in advanced atherosclerosis, we examined imaging data and biopsies from 72 patients who underwent carotid artery ultrasonography and carotid endarterectomy (CEA) (**Figure** **2**a). Staining of atherosclerotic plaque tissues with H&E and antibody targeting SA‐β‐gal to define the relationship between morphological grading in ultrasound, vulnerable plaque score (VPS, according to Table S1, Supporting Information), and SA‐β‐gal positive area (Figure 2b) revealed that SA‐β‐gal expression was significantly positively correlated with atherosclerotic pathological classification by vulnerable plaque score (R^2^ = 0.696, p<0.001, Figure 2d), whereas ultrasonic morphological grading shared an obviously weaker, but still significant, positive correlation with VPS (R^2^ = 0.362, p = 0.002; Figure 2d). In addition, SA‐β‐gal^+^ areas colocalized with α‐SMA^+^ vascular VSMCs and CD68^+^ macrophages. The colocalization of SA‐β‐gal was more general with α‐SMA than with CD68, especially in plaques with higher VPS (Figure 2c). This SA‐β‐gal^+^ α‐SMA^+^ cell population progressively increased with higher instability of atherosclerotic plaques (i.e., VPS; Figure 2e), suggesting a possible active role in plaque progression (R^2^ = 0.72, p<0.001). Although CD68^+^ macrophages in atherosclerotic plaques showed lower accumulation of SA‐β‐gal, colocalization of these signals also gradually increased with plaque progression (Figure 2f, R^2^ = 0.70, *p* < 0.001). These results obtained from clinical biopsies corroborated evidence of SA‐β‐gal upregulation in atherosclerosis from previous reports, while further revealing significantly greater SA‐β‐gal distribution in VSMCs and a linear relationship with plaque progression.

To further validate these findings, we investigated dynamic changes in SA‐β‐gal^+^ cells within atherosclerotic progression in VSMCs lineage‐traced ApoE^‐/‐^ mice (ApoE^−/−^ai9^+^/myh11^cre^) and general ApoE^‐/‐^ mice, and these mice were given a high‐fat diet (HFD) for 0, 4, 12, 16, and 24 weeks to induce varying degrees of atherosclerotic lesions (Figure 2g). We observed that SA‐β‐gal was expressed throughout the entire plaque progression process from 4 to 24 weeks(w), as well as the increase of ai9^+^ VSMCs and MOMA‐2^+^ macrophages (Figure 2h). The percentage of SA‐β‐gal^+^ senescent cells gradually increased, reaching significance at 24 weeks (Figure 2i). We further investigated the colocalization of various senescence markers (SA‐β‐gal, p16, p21) with ai9^+^ VSMCs or MOMA‐2^+^ macrophages. 3D reconstruction imaging revealed that SA‐β‐gal expression was more extensively co‐localized with ai9^+^ VSMCs (Figure 2i,j), as well as with p16 and p21(Figure S1a, Supporting Information). A minor portion of senescence markers (SA‐β‐gal, p16, p21) was observed to co‐localize with MOMA‐2^+^ macrophages (Figure 2i,k; Figure S1b, Supporting Information). In HFD 24w ApoE^−/−^ai9^+^/myh11^cre^ mice, we observed that ≈65.62% of cells expressing senescence markers co‐localized with ai9^+^ VSMCs (Figure 2j). These cells, which were double‐positive for ai9 and senescence markers, were identified as senescent VSMCs. Previous research has shown that a significant number (50–70%) of cells within atherosclerotic plaques, including foam cells, originate from VSMCs.^[^ ^28^ ^]^ The phenotypic transformation of a large proportion of VSMCs into SA‐β‐gal^+^ senescent cells contributes to the expansion of the lipid core and rupture of the fibrous cap.^[^ ^29^ ^]^ These mechanistic findings align with our observation of a high proportion of VSMCs in progressive plaques.

Additionally, SA‐β‐gal activity also increased significantly with plaque progression (Figure S1c,d, Supporting Information) and positively correlated with the percentage of lipid core (Figure S1e, Supporting Information). Additionally, SA‐β‐gal^+^ senescent cells are uniformly distributed throughout the plaques, effectively reflecting the morphological characteristics of these lesions. In naturally aged C57BL/6 mice, aged aorta exhibited no discernible atherosclerotic plaques, and SA‐β‐gal activity staining revealed a uniformly elevated trend compared with normal controls. VSMCs exhibited slightly elevated SA‐β‐gal activity and upregulated expression of senescent molecular markers p16 and p21 (Figure S2, Supporting Information). These cumulative results thus supported a role of SA‐β‐gal expression in plaque development and increased atherosclerotic pathological classification. Monitoring based on the cellular senescence process offers significant advantages for early and continuous surveillance, allowing for simultaneous tracking of both morphological and pathological changes.

### 2.2 Characterization of an SA‐β‐Gal Responsive PPA‐gal‐LNP Photoacoustic Probe

**Figure 3:**
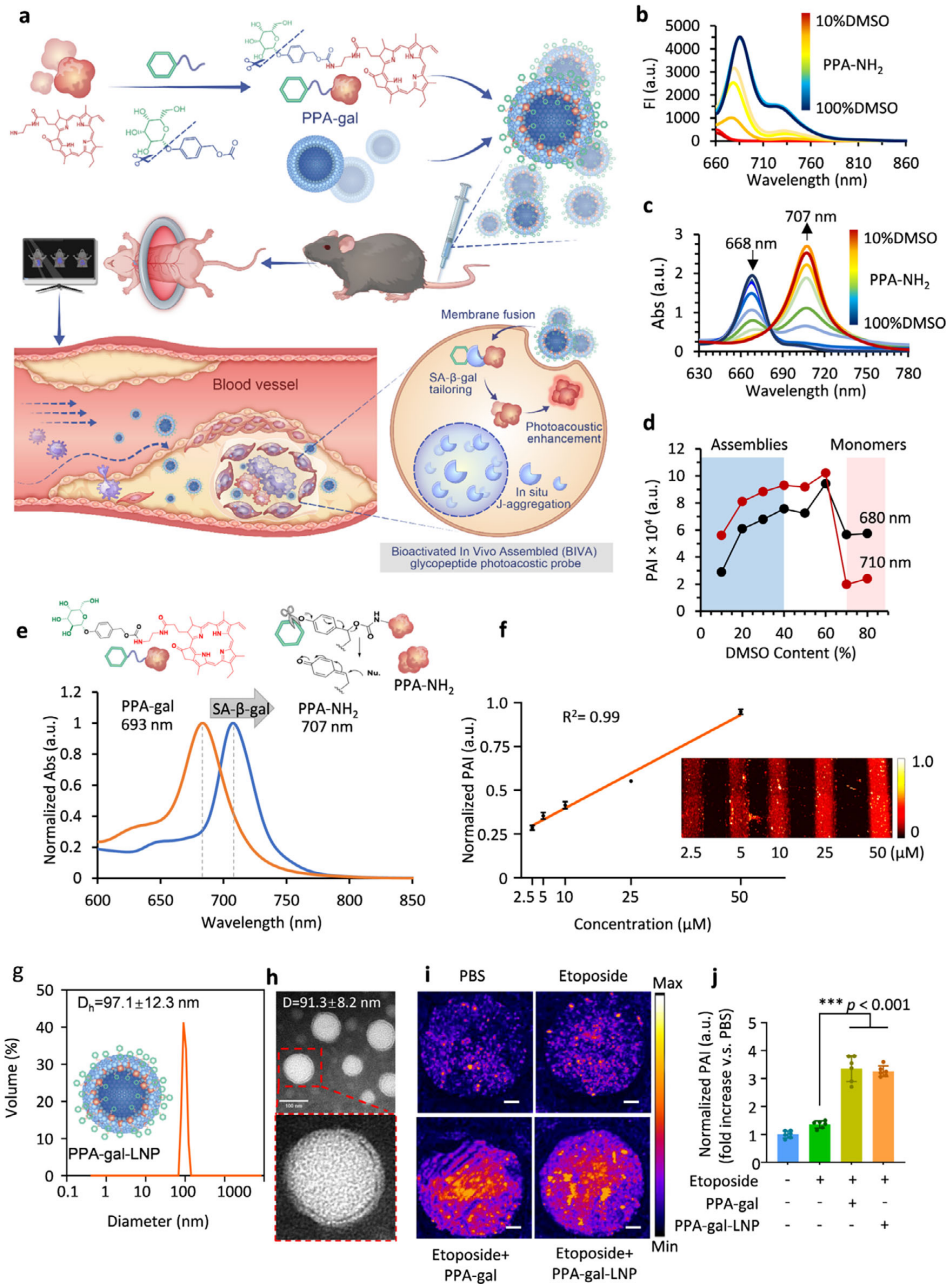
Characteristics of photoacoustic probes. a) Schematic diagram for the composition of PPA‐galactose‐LNP and self‐assembles in cells and becomes a photoacoustic probe in response to β‐galactosidase. b) Fluorescence absorption spectra of PPA‐galactose in different aggregation states. c) UV–vis absorption spectra of PPA‐galactose in different aggregation states. d) Photoacoustic signal of PPA‐galactose in different aggregate states. e) UV–vis absorption spectra of PPA‐galactose and PPA‐NH_2_. f) Linear relationship between the PPA‐galactose‐LNP concentration (2.5 to 50 µmol L^−1^) and the PA_710_ (*n* = 3). g‐h) Particle size distribution and TEM of PPA‐galactose‐LNP. i) Photoacoustic imaging of senescent cells utilizing PPA‐galactose or PPA‐galactose‐LNP. j) Quantitative analysis of variations in photoacoustic signal intensity across cells exhibiting varying degrees of senescence (*n* = 5). Data in j are represented as mean ± s.d. Statistical significance was assessed via a one‐way ANOVA with post hoc Tukey's HSD test performed by GraphPad Prism. Data in f are analyzed by regression analysis performed by SPSS.

Previous studies have shown that the imaging depth of PAI in biological tissues is limited to 2–4 cm. Given that the subcutaneous depth of the carotid artery ranges from 12 to 18 mm and its diameter is 6 to 8 mm, the carotid artery presents an ideal anatomical target for PAI monitoring.^[^ ^30^, ^31^, ^32^, ^33^, ^34^ ^]^ Leveraging the advantages of PAI for carotid atherosclerotic plaque detection, combined with the excellent biosafety profile of the nanoprobe, we employed photoacoustic computed tomography (PACT), which offers deeper tissue penetration (4–5 cm) and high‐quality imaging, making it a promising tool for the clinical monitoring of carotid atherosclerotic plaques.^[^ ^31^, ^35^ ^]^ Considering the biocompatibility and biodegradability of PPA as a PAI signal molecule, it holds a high priority for clinical use.^[^ ^36^, ^37^ ^]^ The peak absorption of PPA in the near‐infrared window (620–950 nm) avoids interference from intrinsic chromophores, thus maximizing tissue penetration depth.^[^ ^38^ ^]^ Thus, to quantitatively image SA‐β‐gal activity in vivo, we developed a chlorophyll‐derived pyropheophorbide‐a (PPA) photoacoustic signal motif responsive to SA‐β‐gal, and employed a liposome‐loaded nanoformulation that provides an optimized hydrodynamic design for efficient accumulation into atherosclerotic plaques through membrane fusion (**Figure** **3**a). This motif could be recognized and cleaved by intracellular SA‐β‐gal at the O‐linked galactose (gal) group, triggering an elimination reaction that results in in situ J‐aggregation of the released PPA (Figure 3a) through a bioactivated in vivo assembly (BIVA) mechanism.^[^ ^39^ ^]^ The detailed synthesis procedure (Figure S3, Supporting Information) and characterization of PPA‐gal are shown in Figures S4 and S5 (Supporting Information).

We examined the fluorescence and UV–vis spectra to define the photoacoustic signal induced by J‐aggregation under a gradient of H_2_O to dimethyl sulfoxide (DMSO) ratio from 0% to 90% (Figure 3b,c). We observed that fluorescence was quenched upon J‐aggregation due to dense molecular stacking that shifted the emission toward heat. At the same time, the absorption spectra exhibited a red‐shift from 668 nm (for monomers) to 707 nm (for J‐aggregates). Photoacoustic monitoring of the monomer and aggregate signals over the course of the J‐aggregation process with cross‐point analysis revealed an inverse relationship in photoacoustic signal intensity between the monomeric and aggregated states (Figure 3d), as described in detail in our previous work.^[^ ^36^ ^]^ This photoacoustic design principle also enabled semi‐quantitative estimation of enzyme activity in vivo.^[^ ^40^ ^]^ To effectively control the aggregation in vivo, we modified the PPA with a galactose functional group to form PPA‐gal, ensuring it is specifically recognized by SA‐β‐gal to catalyze its aggregation. The PPA‐gal chlorophyll derivative exhibited a distinct UV absorption peak at 693 nm (Figure 3e), while the cleaved PPA aggregates showed an absorption peak at 707 nm. In addition, we determined the lower limit of detection and linear detection range of 2.5–50 µmol L^−1^, which showed a good linear fit (R^2^ >0.99; Figure 3f).

To enhance histocompatibility, stability, and circulation time in the bloodstream, the photoacoustic probes, PPA‐gal, were encapsulated in clinically applicable nanoliposomes. We encased the PPA‐gal in lipid nanoparticles to fabricate PPA‐gal‐LNPs (Figure 3g), which had a dynamic hydrated particle size of 97.1±12.3 nm, as determined by dynamic light scattering (DLS). Subsequent transmission electron microscopy (TEM) observation of LNP morphology showed a diameter of 91.3± 8.2 nm (Figure 3h). The PPA‐gal‐LNP exhibited a characteristic phospholipid bilayer vesicle structure and demonstrated relatively high photothermal conversion efficiency and stability (Figure S6a–c, Supporting Information). The pH/enzyme‐responsive release curves of PPA‐gal‐LNPs are shown in Figure S6d,e (Supporting Information). To determine the specific amplification of the photoacoustic signal in senescent cells in vitro, we applied the senescence‐inducing agent, etoposide, to VSMCs and found that senescence levels were proportional to the dosage, and that SA‐β‐gal expression increased along with cell senescence levels (Figure S7a, Supporting Information). Observation by photoacoustic microscope showed that treatment with PPA‐gal or PPA‐gal‐LNP provided a significantly stronger photoacoustic signal of senescent VSMCs than that in cells treated with etoposide alone (Figure 3i,j). To investigate the probe's responsiveness in naturally aging cells, primary vascular smooth muscle cells (VSMCs) were cultured until replicative senescence (passage >35). VSMCs cultured at passages 2–8 served as the normal control. Significantly increased SA‐β‐gal activity was observed in high‐passage VSMCs (>35) compared to controls (see Figure S7b, Supporting Information). CCK‐8 assays confirmed that neither PPA‐gal nor PPA‐gal‐LNP affected the viability of replicatively senescent or normal VSMCs (Figure S7c, Supporting Information). In replicatively senescent VSMCs, treatment with either PPA‐gal or PPA‐gal‐LNP elicited significantly stronger photoacoustic signals compared to those in normal control VSMCs. Additionally, both PPA‐gal and PPA‐gal‐LNP treatment elicited photoacoustic signals that were significantly different from those observed with PBS treatment (Figure S7d,e, Supporting Information). These results collectively indicated that the PPA‐gal‐LNPs could be used for targeted visualization of atherosclerotic plaques by enhancing the photoacoustic signal in vitro.

### 2.3 Ex Vivo PAI Behavior of PPA‐Gal‐LNP in ApoE^−/−^ Mice

**Figure 4:**
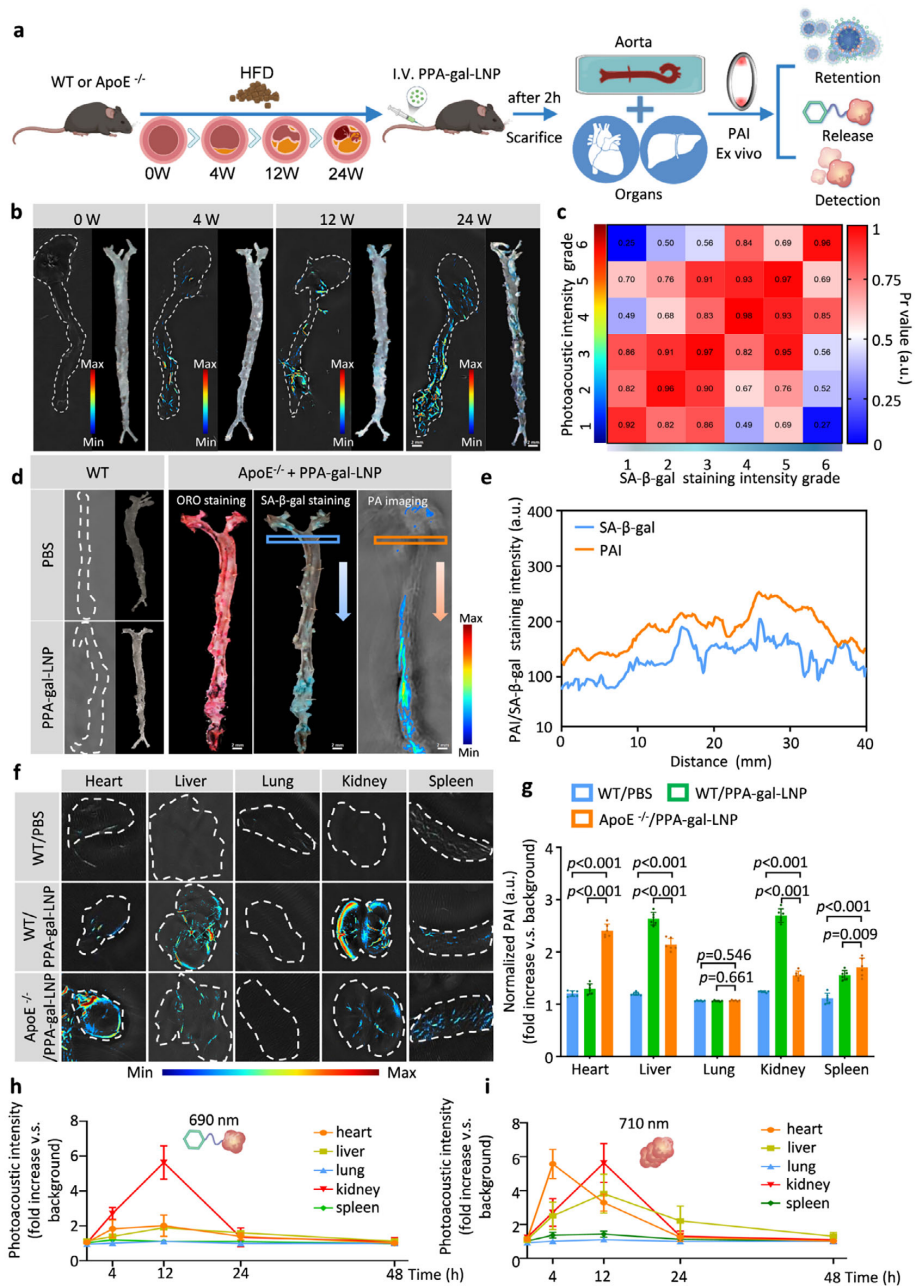
Ex vivo photoacoustic Imaging of aortal atherosclerotic lesions and organ distribution in ApoE^−/−^ mice. a) Scheme of PPA‐gal‐LNP for photoacoustic imaging of atherosclerotic plaques. b) Changes in aorta photoacoustic signal intensity and β‐galactosidase staining during different periods of high‐fat feeding. c) Pearson's correlation analysis of photoacoustic signal intensity and β‐galactosidase staining. (2 mm^2^ per ROI, 3 ROIs per mouse, n  =  3 WT/n  =  5 ApoE^−/‐^ mice) d) Photoacoustic imaging and corresponding galactosidase staining were performed on aortas from different groups (scale bar = 2 mm). e) Plot profile analysis of photoacoustic signal with intensity of β‐galactosidase staining in the marked position shown in (d) (*n* = 3). f‐g) Photoacoustic imaging of different organs and comparison of corresponding quantitative data (*n* = 5, scale bar = 2 mm). h‐i) Different organ photoacoustic signal intensities at different time points in 690 nm (monomers) and 710 nm (polymers) (*n* = 5). Data in g‐i are represented as mean ± s.d. Statistical significance was assessed via a one‐way ANOVA with post hoc Tukey's HSD test performed by GraphPad Prism.

Two hours post‐injection of PPA‐gal‐LNPs via intravenous administration, photoacoustic imaging (PAI) and fluorescence imaging were conducted to assess the intra‐tissue retention, intracellular release, and J‐aggregation of PPA‐gal‐LNPs in the aorta and other organs of both ApoE^−/−^ and wild‐type (WT) mice. The aorta was observed under excitation at 710 nm, and spatial and time‐lapse analyses were performed (**Figure** **4**a). PPA‐gal‐LNPs, formulated with Cy7, demonstrated significantly higher efficiency in targeting plaques, both in the aorta and atherosclerotic lesions, compared to PPA‐gal without LNPs (Figure S8a,b, Supporting Information).

This indicates that the biocompatible LNP surface enhances targeting specificity. Oil Red O (ORO) and H&E staining revealed plaque progression in ApoE^−/‐^ mice subjected to 0–24 weeks of a high‐fat diet (HFD) (Figure S8c,d, Supporting Information). Photoacoustic signals progressively increased as the HFD duration extended (Figure 4b). To quantify the relationship between the intensity of photoacoustic signals and SA‐β‐gal staining in regions of interest (ROIs) in the aorta (2 mm^2^ per ROI, 3 ROIs per mouse), Pearson's correlation coefficients (Pr values) were calculated. High Pr values (Pr ≥ 0.92) indicated strong accuracy of PPA‐gal‐LNP‐based PAI for detecting atherosclerotic lesions (Figure 4c). A distinct difference in photoacoustic signals was observed between 12‐week HFD ApoE^−/−^ mice and WT mice (Figure 4d). The distribution of the photoacoustic‐positive area in the aorta correlated well with the SA‐β‐gal‐positive area (Figure 4d,e). Intensity profiles of the entire aortic wall showed high consistency between photoacoustic signal intensity and SA‐β‐gal activity staining (Figure 4e).

After 12 weeks of HFD, advanced plaques were induced.^[^ ^41^ ^]^ We categorized the HFD timeline into three intervals: 0–4 weeks (negative plaques), 4–12 weeks (positive plaques), and 12–24 weeks (advanced plaques). Accordingly, photoacoustic threshold values corresponding to SA‐β‐gal activity in plaques at different stages were determined: negative plaques (0–40), positive plaques (40–110), and advanced plaques (110–255) (Figure S8e, Supporting Information). These results suggest that the distribution and intensity of PPA‐gal‐LNP‐induced photoacoustic signals in aortic plaques closely matched SA‐β‐gal expression, enabling semi‐quantitative imaging of SA‐β‐gal activity in the plaque microenvironment ex vivo.

Further analysis of the relative distribution of photoacoustic signals across various organs revealed that, in 12‐week ApoE^−/−^ mice, photoacoustic signals were predominantly enriched in the cardiac valves, while WT mice showed a mild increase in signals in the liver and kidney (Figure 4f,g). Dynamic signal attenuation of PPA‐gal‐LNPs was assessed in monomers and polymers at 670 nm or 710 nm in WT mice. These experiments showed that PPA‐gal‐LNPs were predominantly eliminated from the kidney and liver within 24 h (Figure 4h,i). These findings confirm that PPA‐gal‐LNPs are specifically activated at 710 nm in atherosclerotic lesions, including the aorta and cardiac valves, and eliminated from the kidney and liver within 24 h. Moreover, the photoacoustic signal stability of aortic plaques was analyzed within 72 h. The signal intensity at 710 nm gradually decayed after 4 h, with ≈50% still retained at 12 h. It gradually cleared by 24 h and beyond, demonstrating a retention time within vascular plaques not exceeding 24 h (Figure S8f,g, Supporting Information).

### 2.4 Real‐Time Multi‐Wavelength Dynamic PAI of Aortic Atherosclerotic Plaques In Vivo

**Figure 5:**
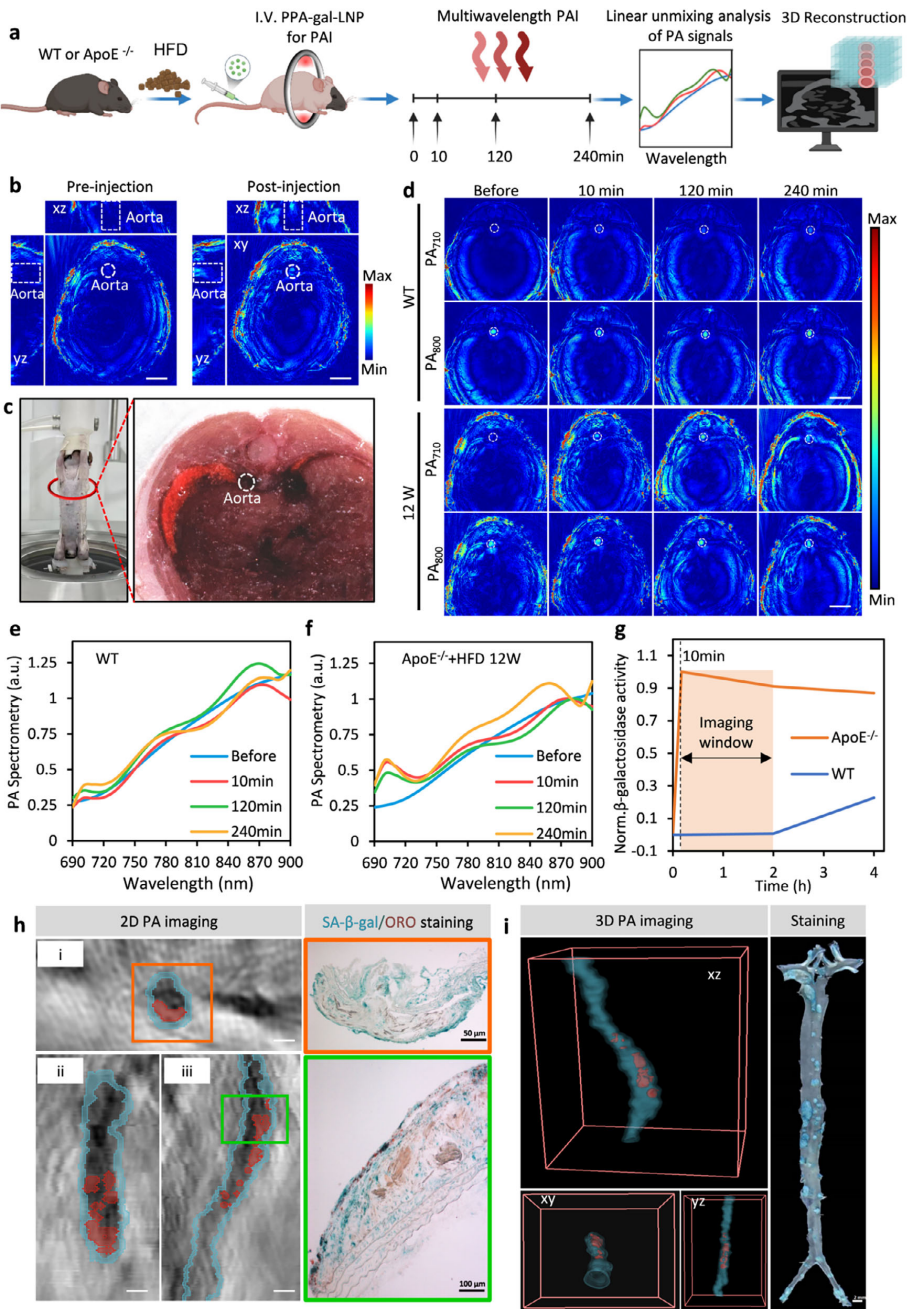
Multi‐wavelength PAI and unmixing analysis of PPA‐gal‐LNP. a) Scheme of PPA‐gal‐LNP for multispectral PAI of atherosclerotic plaques. b) 3D PAI of 12‐week‐old ApoE^−/−^ mice before and after injection of PPA‐gal‐LNP. Excitation: 710 nm. Aorta regions were depicted by dotted circles. c) Cross‐sectional anatomy of mice. Aorta regions were depicted by dotted circles. d) PAI after injection of PPA‐gal‐LNP of WT and 12 W ApoE^−/−^ mice (*n* = 5). Aorta regions were depicted by dotted circles. e‐f) Multispectral Intensity after injection of PPA‐gal‐LNP of WT and 12 W ApoE^−/−^ mice at different time points. g) β‐galactosidase activity measured by photoacoustic intensity at different time points. h) The Grayscale structural image acquired by the PA system reveals the vascular structure and plaque location. PA710/PA800 was employed as the signal for SA‐β‐galactosidase in the plaque, while PA800 served as the vascular signal. Top view of photoacoustic tomography of the aorta and plaques (i), Front view (ii) and Side view (iii). The blue represents the vessel wall, and the red represents the senescent plaques. SA‐β‐galactosidase and ORO staining of corresponding tissue sections. i) 3D visualization of blood vessel walls and aging plaques, the blue represents the vessel wall, and the red represents the senescent plaque. Gross image of the aorta after SA‐β‐gal staining.

Building on the ex vivo results, we further explored the in vivo imaging capability of PPA‐gal‐LNPs for detecting atherosclerotic plaques (**Figure** **5**a). Ten minutes after intravenous injection of PPA‐gal‐LNPs, a notable enhancement of photoacoustic signals was observed under 710 nm excitation (Figure 5b). The region of enhanced signal in vivo corresponded with the anatomical location of the aorta (Figure 5c).

Dynamic photoacoustic images were acquired from both WT healthy mice and 12‐week ApoE^−/−^ mice at 710 and 800 nm (the latter serving as an internal photoacoustic reference) after injection of PPA‐gal‐LNPs (Figure 5d). The aortic position, delineated by the 800 nm signal, exhibited significant enhancement at 710 nm post‐injection in the 12‐week ApoE^−/‐^ mice (Figure 5d). To account for the influence of various factors such as light intensity, tissue scattering, and background absorption on PAI results,^[^ ^24^, ^42^ ^]^ we employed multi‐wavelength real‐time in vivo imaging (670–900 nm) before and after PPA‐gal‐LNPs injection to correct the photoacoustic signal. Considering the relatively indistinct boundaries of plaques within the mouse aorta under photoacoustic tomography, we refrained from segmenting the plaques and measuring the changes of probe signal within the plaque regions. Additionally, due to the relatively weak photoacoustic signals from other biological tissues, including plaques, compared to the hemoglobin and probe signals, and the issue of spectral coloring effect from the deeper location of the aorta within the mouse, we propose a linear unmixing based on biological prior spectral correction (LUBPSC) for effective extraction of the probe signal. Specifically, photoacoustic signal strength within the region of interest (ROI) is obtained after delineating the aortic region based on anatomical priors. Given that the oxygenated hemoglobin content in the artery exceeds 95%, we can calculate the spectral coloring effect based on this prior and photoacoustic signal in the ROI, and subsequently correct the photoacoustic spectra in the ROI post‐probe injection. Following spectral correction, a linear umixing approach is employed to distinguish the hemoglobin signal and probe signal within the ROI, enabling calculation of the probe signal's variation. The mathematical expression of LUBPSC, shown in Figure 5e,f, was calibrated using the method detailed in the Supporting Information Experiment Section.

The data revealed a rapid increase in optical absorption at 710 nm following nanoprobe injection in 12‐week ApoE^−/−^ mice, confirming the PPA‐gal‐LNPs induced specific SA‐β‐gal‐concentration‐dependent absorption at 710 nm. SA‐β‐gal activity was subsequently quantified, and the optimal imaging time window was determined using a linear unmixing method^[^ ^43^ ^]^ (Figure 5g). These results indicate that SA‐β‐gal activity can be accurately quantified, with a significant difference in photoacoustic signal observed between 10 min and 2 h post‐injection. This demonstrates that multi‐spectral correction enables precise in vivo quantification of SA‐β‐gal activity using PPA‐gal‐LNP‐based photoacoustic imaging, with a stable continuous imaging window from 10 min to 2 h. It's worth noting that repeated administering the probe within its retention time window (<24 h for PPA‐gal‐LNP) may exert some influence on the imaging window. Repeated administration often narrows, blurs, or even disrupts the ideal imaging window, primarily due to interference from residual drug (such as increased background signal, among other effects). In this study, our observation indicated that repeated administration at intervals of 24 h or longer has no significant impact on the imaging window.

PAI can achieve cross‐sectional scanning of the common carotid artery, thoracic aorta, and abdominal aorta (Figure S9a,b, Supporting Information).To minimize the distractions caused by skin and hair, we utilized PCSK9‐overexpressed nude mice, which received AAV9‐Pcsk9 i.v. injection and were fed a high‐fat diet for 16 weeks. As shown in the 2D view (Figure 5h), significant signal enhancement was observed in the aortic areas across transverse (XZ), coronal (XY), and sagittal (YZ) planes. Two hours after intravenous injection of PPA‐gal‐LNPs, photoacoustic tomography was performed under excitation ranging from 670 to 900 nm, with 800 nm serving as an internal reference to reduce blood signal interference. The plaque regions by selecting areas with the highest 710 nm/800 nm ratio. Photoacoustic signals observed in the aortic wall plaques were consistent with the pathological staining results (Figure 5h). Notably, continuous scanning of the transverse (XZ), coronal (XY), and sagittal (YZ) planes enabled the reconstruction of 3D images of plaques and the aortic vascular wall (Figure 5i), providing insights into the distribution, morphological features, and pathological progression of plaques.

### 2.5 Dynamic Monitoring of Atherosclerotic Progression Compared with Clinical Imaging Methods

**Figure 6:**
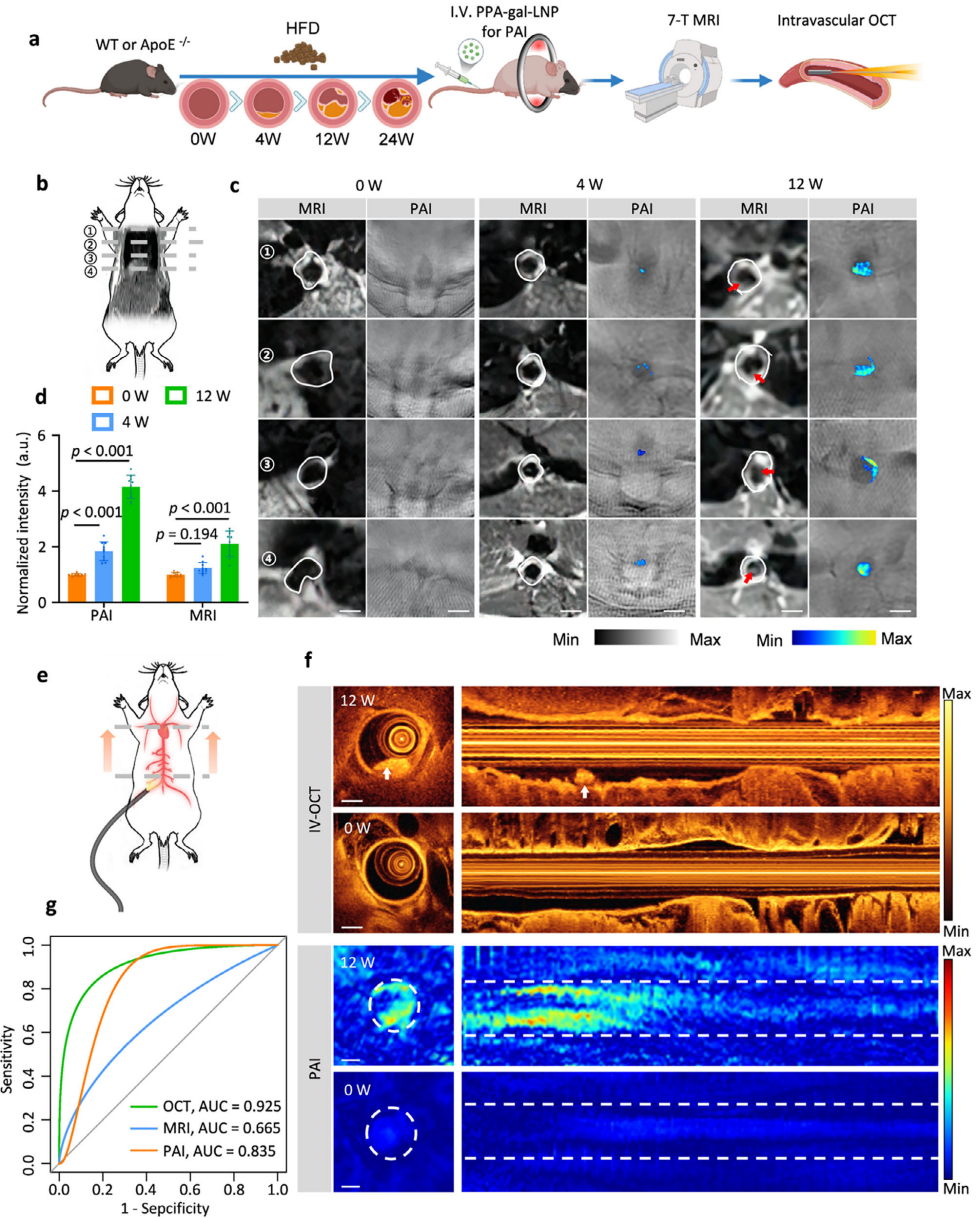
PAI, MR, and intravascular OCT images of plaques. a) Scheme of PAI, MRI, and intravascular OCT (IV‐OCT) for atherosclerotic plaques. b) Schematic diagram of the MRI and photoacoustic tomography site. c) Atherosclerotic plaques of MRI and PAI in ApoE^‐/‐^ mice fed with a high‐fat diet for different weeks. Red arrows indicate atherosclerotic plaque location. d) Comparison of magnetic resonance and photoacoustic signal intensity at atherosclerotic plaques (scale bar = 1 mm, *n* = 5). e) Schematic diagram of IV‐OCT. f) 2D IV‐OCT cross sections and longitudinal scan of the mouse aorta (top). White arrows indicate atherosclerotic plaque. PAI cross sections and longitudinal reconstruction of the mouse aorta (bottom). The white dotted line indicates the mouse aorta. g) Receiver operating characteristic (ROC) curve was constructed on the sensitivity and specificity of photoacoustic imaging, magnetic resonance imaging, and IV‐OCT for detecting atherosclerotic plaques (scale bar = 0.4 mm). n  =  53 independent aortic regions (n  =  27 normal vascular wall /n  =  26 plaque) in n  =  21 biologically independent animals (n  =  6 WT/n  =  15 ApoE^−/−^ mice). Data in d are represented as mean ± s.d. Statistical significance was assessed via a one‐way ANOVA with post hoc Tukey's HSD test performed by GraphPad Prism. Data in g are performed by ROC analysis with Bonferroni correction.

High‐resolution MRI has been utilized to detect plaque vulnerability, and OCT exhibits unparalleled in its ability to detail plaque morphology and offers the highest specificity and imaging performance.^[^ ^44^, ^45^ ^]^ To further assess the sensitivity and specificity of PPA‐gal‐LNP‐based photoacoustic imaging, we utilized 7T magnetic resonance imaging (MRI) and intravascular OCT in conjunction with PAI. ApoE^−/‐^ and WT mice underwent PAI, MRI, and OCT in sequence, with less than a one‐day interval between each imaging modality (**Figure** **6**a). Cross‐sectional images of plaques obtained from the same scans were compared (Figure 6b). The results demonstrated that PPA‐gal‐LNP‐based photoacoustic imaging provided enhanced photoacoustic signals after 4 weeks, while T1‐weighted MRI imaging revealed plaque signals only at 12 weeks (Figure 6c,d). Furthermore, PPA‐gal‐LNP‐based photoacoustic imaging not only aligned with conventional morphological MRI scans at 12 weeks but also provided higher contrast (Figure 6c). These findings indicate that PAI can detect plaques at earlier stages than high‐resolution MRI and offers a more detailed representation of plaque pathological changes in vivo.

In comparison with intravascular OCT (Figure 6e), which excels in providing superior morphological details of plaques, SA‐β‐gal‐responsive PAI offers comparable morphological features and cross‐sectional positional information while highlighting senescence‐related pathological changes in both the plaques and surrounding vascular walls (Figure 6f). To evaluate the performance of each imaging method, we generated receiver operating characteristic (ROC) curves and calculated the area under the curve (AUC) as a performance indicator. Intravascular OCT achieved the highest AUC value (OCT: AUC = 0.925, 95% CI: 0.854‐0.995. PAI: AUC = 0.835, 95% CI: 0.720‐0.949. MRI: AUC = 0.665, 95% CI: 0.515‐0.814) among the three imaging techniques based on 53 independent aortic regions (n  =  27 normal vascular wall /n  =  26 plaque) in n  =  21 biologically independent animals (n  =  6 WT/n  =  15 ApoE^−/−^ mice) (Figure 6g). PAI, however, demonstrated the highest sensitivity (0.962). These results underscore that, as a non‐invasive technique, PPA‐gal‐LNP‐based photoacoustic imaging (PAI) offers higher sensitivity and specificity than MRI, with a superior detection rate when compared to OCT.

While MRI is sensitive to intraplaque hemorrhage, it remains challenging in accurately mapping fibrous cap thickness and lipid core size.^[^ ^44^, ^45^ ^]^ Moreover, MRI is not universally accessible due to the large and expensive imaging equipment required, whereas photoacoustic imaging (PAI), in conjunction with ultrasound, offers a more broadly applicable alternative.^[^ ^46^ ^]^ Our current study demonstrates that SA‐β‐gal‐responsive PAI provides a faster, more affordable, and earlier imaging technique compared to MRI, offering the potential for full, dynamic, real‐time pathological imaging of plaque progression. Identifying key time points for the initiation and rapid progression of plaques is crucial for clinical decision‐making regarding drug administration and interventional therapies,^[^ ^7^ ^]^ and our findings could help guide such medical decisions. While several challenges remain for the clinical application of this study, particularly in achieving higher quantification accuracy in PAI.^[^ ^31^ ^]^ The method holds promise for broader use. One consideration is that PAI can be less effective for patients with darker skin tones, although it remains widely applicable to individuals with fair or yellow skin tones. In comparison to intravascular OCT, PPA‐gal‐LNP‐based PAI excels in identifying senescence‐related pathological features within plaques and the surrounding vascular wall, offering superior sensitivity. It is essential to recognize the limitations of OCT, an invasive procedure that carries inherent risks. OCT should be used cautiously in patients with only one remaining vascular access, severely impaired renal function, or a known allergy to contrast agents. Complications, including catheter‐related issues, have been reported in up to 0.6% of cases.^[^ ^47^ ^]^ Furthermore, OCT is expensive due to the need for disposable materials. Therefore, PAI's advantages include being non‐invasive, cost‐effective, and widely applicable. However, there are still some limitations, this study focused on validating PAI against structural modalities (MRI/OCT). Direct comparisons with functional techniques like PET/CT and NIRF in identical models represent an important future direction. For deep plaque imaging, particularly in coronary arteries, intravascular ultrasound (IVUS) remains the clinical standard. IVUS offers significant advantages, including a large field of view for plaque structural assessment, deep tissue penetration, and blood‐insensitive imaging. While transcutaneous photoacoustic imaging yields suboptimal results for such applications, intravascular photoacoustic imaging (IVPA) presents distinct advantages. Crucially, IVPA surpasses IVUS through its revolutionary capacity for molecular and functional characterization. This enables depth‐resolved compositional analysis of atherosclerotic plaques, vulnerability assessment, and quantitative evaluation of therapeutic responses (device‐based, pharmacological, and lifestyle interventions) with high molecular specificity. However, IVPA limitations include signal attenuation by blood (requiring clearance techniques) and reduced entire visualization efficacy in plaques with extensive lipid cores.

### 2.6 Cellular Specificity and Biological Safety of the SA‐β‐Gal Responsive Probe

To further investigate the specificity of the nanoprobe on SA‐β‐gal^+^ cells in vivo, we examined the correlation between photoacoustic signal intensity and the population of SA‐β‐gal^+^ cells. Interestingly, the distribution of SA‐β‐gal^+^ α‐SMA^+^ cells closely mirrored the areas of enhanced photoacoustic signal in the PAI tomography images, two hours post‐injection of PPA‐gal‐LNPs (Figure S9c, Supporting Information). The percentage of enhanced photoacoustic signal area in the aorta correlated positively with the percentage of the SA‐β‐gal^+^ α‐SMA^+^ population area (R^2^ = 0.69, *p* < 0.001, Figure S9d, Supporting Information).

To confirm the probe's specificity for senescent cells in vitro, we employed cultured macrophages with high lysosomal content (Figure S10a, Supporting Information). We compared SA‐β‐gal activity and photoacoustic signal intensities between normal and senescent macrophages. The results revealed significantly higher SA‐β‐gal activity and photoacoustic signal intensity in senescent macrophages compared to normal macrophages (Figure S10b, Supporting Information). Given that SA‐β‐gal exhibits high activity in both the cytoplasm and lysosomes under suboptimal pH conditions, it serves as a unique biomarker to distinguish between normal and senescent cells.^[^ ^48^, ^49^ ^]^ To further explore this, we isolated the cytoplasm and lysosomes from macrophages and measured photoacoustic signal intensities in these compartments for both normal and senescent cells. Notably, senescent macrophages showed a marked enhancement of photoacoustic signals in both the cytoplasm and lysosomes, whereas normal macrophages exhibited limited signal accumulation, primarily in the lysosomes (Figure S10c, Supporting Information).

We also assessed cell viability after PPA‐gal‐LNPs treatment. The PPA‐gal‐LNPs demonstrated minimal cytotoxicity, maintaining the integrity of both RAW264.7 macrophages, VSMCs, and primary aortic endothelial cells (Figure S10f–h, Supporting Information). Furthermore, no significant intimal vascular injury was observed (Figure S11a, Supporting Information). Subsequently, mice were administered a high dose of PPA‐gal‐LNPs, and over eight weeks, no significant changes in behavior or weight loss were observed, indicating good health. Additionally, long‐term toxicity was detected with a comprehensive 8‐week follow‐up. Blood cell counts remained unaffected, suggesting that PPA‐gal‐LNPs did not induce long‐term immune responses or cause hematopoietic defects (Figure S11b, Supporting Information). Liver and kidney function tests showed normal hepatic and renal function (Figure S11d, Supporting Information). Histopathological examination of the heart, liver, spleen, lung, and kidney post‐mortem revealed that these organs remained in good condition, unaffected by the high intravenous dose of PPA‐gal‐LNPs (Figure S11c, Supporting Information). Additionally, we performed ORO staining to assess the effects of the LNPs on the lipid accumulation effect on the liver. No increase in lobular lipid accumulation was observed at the 8‐week time point (Figure S11c, Supporting Information). To access repeated‐dosing acute toxicity, we conducted three consecutive doses (10 mg kg^−1^) administration within 72‐h period followed by a 7‐day observation period. PPA‐gal‐LNP demonstrates a favorable safety profile upon repeated dosing, with preserved structural integrity and physiological function across major organ systems (Figure S11e,f, Supporting Information).

To date, near‐infrared auto‐photoacoustic (NIRAPA) imaging has been reported to detect plaque components and differentiate stable from vulnerable plaques in fresh samples,^[^ ^46^ ^]^ raising the possibility of clinical detection based on NIRAPA signals. However, whether in vivo imaging of naturally occurring near‐infrared markers is feasible remains to be further confirmed. Contrast agents help overcome background absorption and function as “smart” (or “activatable”) agents, triggering a prescribed signal change, such as a shift in the peak absorption wavelength, and facilitating dynamic real‐time responses to processes such as enzymatic activity.^[^ ^38^ ^]^ Previously, various nanomaterials have been explored as PAI contrast agents.^[^ ^10^, ^50^ ^]^ For example, VCAM‐1‐targeted or untargeted gold nanomaterials (AuNPs) have demonstrated PAI signals in atherosclerotic rabbit models or ApoE^−/−^ mice.^[^ ^51^, ^52^ ^]^ Additionally, single‐walled carbon nanotubes (SWNTs) enabled the imaging of inflamed arterial plaques using PAI technology with high contrast.^[^ ^10^, ^50^ ^]^ Clinical contrast agents like indocyanine green (ICG) also exhibit aggregation‐induced spectral shifts, forming J‐aggregates that red‐shift their absorption peaks to 810–900 nm. However, >98% of free ICG binds to albumin and lipoproteins upon injection, limiting imaging utility due to its ultrashort plasma half‐life (2–4 min). By contrast, free PPA‐galactose and PPA‐gal‐LNP nanoparticles demonstrate higher stability in vivo, the LNPs provide stable imaging throughout a 10‐min to 2‐h time window and are largely cleared within 24 h. While these studies highlight the potential of PAI, it remains uncertain whether early, dynamic, real‐time monitoring based on these pathological processes can be achieved. Furthermore, the biocompatibility of these probes in vivo, specially AuNPs and SWNTs, requires more systematic evaluation through long‐term toxicity studies for individual nanostructures.^[^ ^31^, ^38^ ^]^ Our nanoprobe is designed based on clinical findings that demonstrate a positive correlation between elevated levels of the senescence marker SA‐β‐gal and atherosclerotic pathological classifications in samples from 72 patients undergoing carotid artery ultrasonography and carotid endarterectomy (CEA). Monitoring cellular senescence provides a unique approach to identifying the onset and progression of plaques. Unlike traditional probes that target single molecular markers, our nanoprobe captures the multi‐stage mechanisms of cellular senescence in plaques, allowing imaging from the earliest stages of plaque formation. As plaques develop, signal intensity progressively increases, offering a dynamic enhancement that provides a comprehensive view of plaque progression, rather than focusing solely on isolated phases.

## 3 Conclusion

This study introduces a noninvasive imaging modality for the quantitative detection of plaque SA‐β‐gal activity as a biomarker in the formation and expansion of atherosclerosis. The photoacoustic nanoprobe, PPA‐gal‐LNP, specifically targets the senescent cell marker SA‐β‐gal, triggering the cleavage of the O‐linked galactose group and the subsequent in situ J‐aggregation of PPA. This aggregation induces a shift to the red emission spectra of PPA, resulting in an enhanced photoacoustic signal at 710 nm, which allows for the in vivo detection of SA‐β‐gal activity. These J‐aggregates improve photostability, reduce exocytosis, and extend the detection window while maintaining a high signal‐to‐noise ratio. The multi‐wavelength PAI, combined with linear unmixing calculations, precisely confirmed the signal specificity of PPA‐gal‐LNP at 710 nm, thereby demonstrating this SA‐β‐gal‐responsive strategy as a specific, dynamic, and quantitative monitoring tool. Additionally, 3D PAI reconstruction showcased the exciting potential for integrated clinical‐morphological and pathological monitoring of multiple plaques within the vascular wall. Thus, the PPA‐gal‐LNP‐based PAI presents an early, accurate, and noninvasive clinical tool for in vivo monitoring of plaque formation and expansion by responding to essential cellular processes during atherosclerotic progression. This approach presents an exciting opportunity for the safe and efficient detection of atherosclerosis, requiring only non‐invasive, low‐cost, and widely accessible PAI imaging. We hope that PPA‐gal‐LNP‐based photoacoustic imaging will help transition plaque detection from traditional morphological diagnosis to early‐stage, real‐time pathological screening, enabling clinical decisions to be made before plaque progression accelerates and ischemic symptoms arise.

## 4 Experimental Section

### Clinical Data Collection

The study protocol involving the collection of clinical specimens was granted ethical approval by the Ethics Committee of Wuhan Union Hospital (Wuhan, China, 2018IEC‐J034). A cohort of 70 patients who underwent carotid endarterectomy (CEA) at Wuhan Union Hospital between March 2019 and December 2022 was included, comprising a total of 72 plaques. This cohort consisted of 60 male and 18 female participants. Informed consent was obtained from all patients prior to their participation in the study. Prior to the surgical interventions, each patient underwent a standardized carotid artery ultrasound examination. During this procedure, the internal echogenicity and plaque thickness on the targeted side were meticulously documented. Additionally, parameters such as luminal constriction and the degree of narrowing were evaluated and recorded. The plaques were classified based on their echogenicity into three categories: hypoechoic, mixed echogenic, and hyperechoic. Specifically, hypoechoic plaques were assigned a classification of “1”, mixed echogenic plaques were designated as “2”, and hyperechoic plaques were labeled as “3”.

The definition of vulnerable plaque encompasses five major criteria and five minor criteria (Table S1, Supporting Information). A quantification mechanism was developed, assigning a score of 1 point for each major criterion and 0.5 points for each minor criterion to plaques. Pathological findings served as the reference standard for evaluating plaque stability. Relevant pathological data pertaining to patients' plaques were retrieved from the Hospital Information System (HIS), after which the plaque instability score was re‐assessed.

### Immunofluorescence and Immunohistochemistry

Immunofluorescence or immunohistochemical staining was performed using antibodies against galactosidase (rabbit, 1:100, ab9361, Abcam), smooth muscle actin (a marker for smooth muscle cells, rabbit, 1:100, ab7817, Abcam), CD68 (a marker for macrophages, rabbit, 1:100, ab303565, Abcam), and MOMA‐2 (a marker for macrophages, rabbit, 1:100, ab33451, Abcam) to enable comprehensive analysis of tissue samples. Quantitative evaluations were conducted via statistical analysis of the stained regions, with particular emphasis on quantifying β‐galactosidase‐positive areas within the plaques. Additionally, co‐localization analysis of immunofluorescent staining for galactosidase, smooth muscle actin (SMA), and macrophages (CD68 and MOMA‐2) was performed. The visualization of protein localization within cells was achieved employing a high‐resolution digital fluorescent microscope (Leica DMI600). The evaluation of co‐localization and the determination of the percentage‐positive area were executed using ImageJ software. For confocal z‐axis stacks, images separated by 0.2 µm along the z‐axis were acquired. To ensure standardized fluorescence intensity assessment across experimental iterations, meticulous optimization and maintenance of image acquisition parameters, intensity amplification, and image black levels were uniformly upheld throughout all conducted experiments.

### Synthesis of Nanocarriers

The nano‐photoacoustic probe consists of two components: a photoacoustic probe and a liposome, which together form the complete nano‐photoacoustic probe. Detailed synthetic procedures can be found in the Supporting Information. Briefly, an organic acylation reaction was employed to conjugate the galactose moiety with pyropheophorbide‐a, resulting in the formation of the photoacoustic probe PPA‐galactose. Subsequently, the liposome (LNP) and the photoacoustic probe were integrated using microfluidic technology to produce the nano‐photoacoustic probe (PPA‐galactose‐LNP).

Hydrodynamic size was measured by a dynamic light scattering instrument (Brookhaven Instruments, Holtsville, NY, USA) using deionized water as the dispersant. The morphology of NPs was imaged using a TEM (Tecnai G2‐20). The NPs were dispersed in deionized water to prepare a very dilute suspension (0.1 wt%); a drop of the suspension was then placed onto a 300 mesh carbon‐coated copper grid and dried at room temperature. NPs were negatively stained using phosphotungstic acid (2.0 wt%) and allowed to dry before TEM observation.

### Fluorescence and UV–Vis Spectra of PPA‐NH_2_

The absorption and fluorescence spectra were recorded at 25 °C using an F‐280 fluorescence spectrophotometer and a Cary100Bio UV–vis spectrophotometer with a 10 mm optical path length. PPA‐NH_2_ was dissolved in different DMSO/water mixtures (ranging from 1% to 100% DMSO) by vortexing, achieving a final concentration of 130 µM, followed by a resting period of 10 min before conducting the spectrophotometric analysis. The PPA‐gal samples were subjected to SA‐β‐gal treatment in Tris buffer (20 mM Tris·HCl, 50 mM NaCl, 5 mM CaCl_2_, 0.05% Brij‐35, pH 7.4) at a temperature of 30 °C for a duration of 1 h. Subsequently, the SA‐β‐gal‐treated PPA‐gal samples were analyzed using UV–vis spectroscopy.

### PA Intensity of PPA‐NH_2_

The PA signal was recorded at 25 °C using an MSOT (128 Multi‐Spectral Optoacoustic Tomography) system under a detecting window at 680 and 710 nm. PPA‐NH2 was dissolved in different DMSO/water mixtures (ranging from 1 to 100% DMSO) with a final concentration of 200 µmol L^−1^ by vortexing, followed by a 10‐min incubation period before analysis. The PA data was calculated based on the average value of the cross‐sectional signal of the sample.

### Photoacoustic Imaging of Probe and Cells

The synthesized photoacoustic probes were diluted to various concentrations and introduced into the transparent tube. Subsequently, the photoacoustic image acquisition system, developed by Union Photoacoustic Technologies Co., Ltd., was employed to collect the photoacoustic signals of the probes at different concentrations, utilizing a laser emission wavelength of 710 nm. Finally, a linear fit was applied to the acquired data. Expose the VSMCs to the photoacoustic probe and the nano‐photoacoustic probe, then incubate the senescent VSMCs for 2 h. Subsequently, collect the cells and inoculate them onto a custom‐made cell‐loading well with a diameter of 4 mm. Utilize a 710 nm laser to emit the light wavelength, and collect the photoacoustic signal from the cells. The identical probe and culture duration were employed for the detection of photoacoustic signals in RAW246.7 macrophages.

### Lysosome Isolation and Enrichment

Lysosomes were isolated from RAW246.7 macrophages using the Lysosome Enrichment Kit (Thermo Fisher Scientific, catalogue number #89839) according to the manufacturer's instructions. Macrophages were grown to 80–90% confluence and harvested. ≈100 mg of RAW246.7 macrophages were treated with pre‐chilled Lysosomal Reagent A and lysed by sonication. Lysosomal Reagent B was then added, and the homogenate was centrifuged to remove cellular debris. The clarified supernatant was then applied to a discontinuous gradient of OptiPrep Cell Separation Matrix, followed by ultracentrifugation to purify and enrich for lysosomal fractions.

### Multi‐Wavelength Imaging and Spectral Unmixing

For the photoacoustic imaging procedure, a commercially available small animal photoacoustic imaging system (Union Photoacoustic Technologies Co., Ltd, China) was utilized. The mouse was positioned vertically within a circular scanner that was filled with water. Photoacoustic tomography was conducted on the aorta of each mouse, resulting in the acquisition of a total of 24 spectral measurements within the wavelength range of 670 to 900 nm, with intervals of 10 nm. Mice were anaesthetized, sacrificed, and organs of interest were isolated and embedded in low melting point agarose (2%). PA images were recorded at 710 nm for organs.

For imaging and unmixing, the autocorrelation method of preceding and succeeding frames was employed to eliminate data artifacts caused by mice respiration and to mitigate breathing‐related distortions. Given that the laser exhibits varying light energies across different wavelengths, and considering the differential absorption coefficients of the optical path, coupling medium, and mice at these wavelengths, it was necessary to correct the photoacoustic signal intensity values accordingly. In the absence of probe injection, the predominant signal observed in the photoacoustic image corresponds to oxygenated hemoglobin. Consequently, the photoacoustic signal values from the aortic region, prior to the administration of injections in both groups, were utilized to normalize the photoacoustic signals recorded at various time points throughout the experiment. Following this normalization, high‐order polynomial fitting was employed to mitigate spectral noise. Subsequently, the linear unmixing method was applied to determine the concentration of the probe in the two datasets.

To locate the mouse aorta, anatomical knowledge was applied with threshold segmentation in photoacoustic signals to delineate regions of interest (ROIs) within the aortic region. The spectral coloring effect was calculated based on this prior and photoacoustic signal in the ROI, and subsequently corrected the photoacoustic spectra in the ROI post‐probe injection. First, autocorrelation methods were applied to mitigate respiratory motion artifacts during imaging. Next, a thresholding method was used at 800 nm to segment photoacoustic signals corresponding to the aorta in likely arterial regions. Since body position and location remained consistent across wavelengths, photoacoustic signal intensity was extracted from these regions at other wavelengths. Given the probe's absorption peak at 710 nm, plaque regions were identified by selecting areas with the highest 710 nm/800 nm ratio. The photoacoustic spectra were refined by employing a linear unmixing approach to quantify probe expression precisely. After delineating and spectral correction, probe signals from the designated regions were extracted using a linear unmixing approach, effectively distinguishing the probe signal through this method. PA710 and PA800 images were collected by the PA imaging system, and the PA710/PA800 ratio images were obtained for 3D reconstruction. The 3D‐slicer software (http://www.slicer.org, Surgical Planning Laboratory, Harvard University, Boston, MA, USA, version 4.12.1) was used to perform reconstruction of the tomographic data to obtain 3D images of blood vessels and atherosclerotic plaques with photoacoustic signals.

### Correlation Coefficient Matrix of Photoacoustic Imaging

The isolated aorta underwent photoacoustic imaging and β‐galactosidase staining. The aorta was segmented into six levels based on photoacoustic intensity and similarly into six levels according to the depth of β‐galactosidase staining. Measurements were obtained from 100 mm^2^ rectangular regions on both the photoacoustic and β‐galactosidase images. Subsequently, a correlation coefficient matrix was constructed to analyze the relationship between photoacoustic imaging intensity and β‐galactosidase staining depth.

### Mouse Experiments

All animal protocols were approved by the Medical Ethics Committee of Tongji Medical College and the Institutional Committee of Animal Care and Use, Huazhong University of Science and Technology, Wuhan, China (Wuhan, China, TY20220131). For PA imaging of plaque‐bearing mice, ApoE^−/−^ mice were fed with a high‐fat diet for 4–24 weeks (16% fat and 1.25% cholesterol) to induce atherosclerosis. The mice were divided into two groups (*n* = 5) for photoacoustic imaging in vivo: i) Healthy mice, ii) Plaque‐bearing mice (24 weeks HFD fed). Mice in different groups were intravenously injected with PBS containing PPA‐galactose‐LNP, and PA images were recorded at 670–900 nm at different time points. ApoE^−/−^ai9^+^/myh11^cre+^ mice were used for immunofluorescence colocalization. To evaluate the specificity of the probe to replicative senescent VSMCs, 24‐month‐old ai9^+^/myh11^cre+^ mice (C57BL/6 background) were used as a natural aging model, with 8‐week‐old Myh11‐ai9^+^/myh11^cre+^ mice serving as young controls.

### Establishment of a Cellular Senescence Model

VSMCs were isolated from the aortic tissues of 8‐week‐old C57BL/6 mice and subsequently used to establish an in vitro senescence model. The cells were uniformly seeded onto culture dishes and exposed to different concentrations of etoposide (0, 5, 10, and 20 µM) for a period of 48 h. Following this treatment, the medium was replaced, and the cells were cultured for an additional three days. Senescence‐associated β‐galactosidase (SA‐β‐gal) staining was performed using a kit from Beyotime, and the number of blue‐stained senescent cells was quantified using an inverted microscope. This experiment was conducted in triplicate senescence‐associated β‐galactosidase staining.

Cellular senescence was evaluated by detecting β‐galactosidase activity using a SA‐β‐gal staining kit (Beyotime, Shanghai, China), following the manufacturer's protocol. In summary, cells were fixed with a fixative solution for 15 min at room temperature. Subsequently, the cells were rinsed with PBS and incubated with freshly prepared staining solution overnight at 37 °C. The proportion of positively stained cells (blue cells) relative to the total cell count was determined across six randomly selected microscopic fields. Images were captured at 100 × magnification using a Nikon Eclipse TS100 microscope (Nikon, Japan). SA‐β‐gal activity was additionally assessed in tissue samples. Specifically, aorta specimens or sections obtained from mice were fixed for 10 min, stained for 24 h, and subsequently examined using light microscopy.

### ORO Staining

Mouse aortic and liver tissue sections were fixed in 4% paraformaldehyde (PFA) and then washed three times in PBS. The sections were then stained with an Oil Red O (ORO) solution at room temperature for 30 min. The stained sections were visualized and imaged using optical microscopy.

### MRI

Magnetic resonance scans were performed after 4 or 12 weeks of high‐fat feeding (7‐T Bruker Biospec small animal MRI). The following three sequences were applied: T2, T1, and enhanced T1 as previously described.

### In Situ Mouse Intra‐Aortic OCT Imaging

Mice were humanely euthanized under anesthesia. Saline flushes were administered through a 22‐gauge catheter inserted into the apex of the left ventricle to facilitate in situ pressure perfusion at 160 mmHg during optical coherence tomography (OCT) imaging. A 2.6 Fr OCT imaging wire (HC100, Horimed Technology Co., Ltd, Tianjin, China) was advanced from the abdominal aorta to the top of the aortic arch. Imaging was conducted using a time‐domain OCT system (GEMISIGHT, Horimed Technology Co., Ltd, Tianjin, China) equipped with a near‐infrared light source. Prior to image acquisition, the aortic bifurcation and distal abdominal aorta were secured with silk thread. Images were captured at a pullback speed of 18 mm s^−1^ and a frame rate of 180 frames s^−1^.

### Biocompatibility Safety

For in vitro cytotoxicity assays, macrophages, VSMCs, and primary endothelial cells were seeded into 96‐well plates. Post‐treatment with etoposide, the cells were classified into senescent and non‐senescent groups. Subsequently, these cells were exposed to a gradient of PPA‐galactose‐LNP concentrations, ranging from 0 to 200 µg mL^−1^, over a 24‐h period. The experimental protocol included the addition of 10 µL of CCK‐8 solution and 100 µ L of culture medium to each well. Following a 1‐h incubation, absorbance was measured at 450 nm.

The C57BL/6 mice were systematically allocated into three experimental groups (*n* = 5/group): PBS control, PPA‐galactose, and PPA‐galactose‐LNP (10 mg kg^−1^). Two distinct dosing regimens were implemented: (i) intensive consecutive dosing (10 mg kg^−1^/day for 3 days) followed by a 7‐day observation period, and (ii) longitudinal single‐dose exposure (10 mg kg^−1^) with comprehensive 8‐week follow‐up. Post‐treatment, all animals underwent daily body weight measurements, weekly food and water intake monitoring, and detailed behavioral assessments. Terminal tissue collection was performed for multi‐organ (heart, liver, kidney, spleen, lung) histopathological evaluation using Hematoxylin and Eosin (H&E) staining. To assess the long‐term lipid retention effect of nanomaterial drug delivery, lipid accumulation in liver cells was systematically evaluated using quantitative ORO staining.

Immunohistochemical staining of endothelial cells using CD31 was employed to assess the impact of the probe on vascular endothelium. Additionally, blood samples were collected from mice for comprehensive analysis, including complete blood count, serum lipid profiling, liver function markers, and renal function markers.

### Statistical Analysis

All presented data were expressed as the mean ± standard deviation (SD). The quantity of replicates employed for each experiment and subsequent statistical analysis was explicitly specified in the figure legend. The statistical evaluations were executed through the utilization of GraphPad Prism 8.0 software (GraphPad, San Diego, CA, USA) or SPSS Statistics 25 (SPSS Incorporation, Chicago, IL). The normality of distribution was assessed using the Shapiro‐Wilk test. For statistical comparisons involving more than two groups, a one‐factor analysis of variance (ANOVA) was performed, followed by a Tukey's post hoc test to account for multiple‐group comparisons. Classical univariate ROC (receiver operating characteristic) curve analysis with Bonferroni correction was performed to generate the ROC curve. Statistical significance was defined as p < 0.05. Sample sizes were chosen according to standard guidelines. Number of animals was indicated as “‘n.”’ The required sample size was estimated for 80% power and 95% confidence interval (CI) (2‐sided) using the power calculations appropriate for AUC testing using PASS statistical software.

## Conflict of Interest

The authors declare no conflict of interest.

## Author Contributions

Y.F.Z., H.Y., and H.D.D. contributed equally to this work. B.H., Y.F.Z., and L.L.L. conceived and designed the project. L.L.L. and H.W. carried out the synthesis and characterization of the BIVA probes. H.Y., H.D.D., and C.M. contributed the PAI imaging and 3D reconstruction. F.Z., J.H.W., and H.J.J. performed the fluorescence imaging studies both in vitro and in vivo. J.J.J., Y.N.L., and X.D.S. performed biological security studies. Y.F.Z., Y.H., and C.M. co‐wrote the manuscript. All authors interpreted data, provided critical insights, and edited the manuscript.

## Supporting information



Supporting Information

## Data Availability

The data that support the findings of this study are available from the corresponding author upon reasonable request.
