# Supplementary material for: Real‐Time Monitoring of Atherosclerotic Plaque Using β‐Galactosidase‐Activated Photoacoustic Tomography
Source: Small. 2025 Sep 12;21(44):e06731. doi: 10.1002/smll.202506731 (PMC12590527; doi:10.1002/smll.202506731)
Supplement: Supplementary file 1 — Supporting Information [file SMLL-21-e06731-s001.docx]

**Supporting Information**

**Real-time monitoring of** **atherosclerotic plaque using β-galactosidase-activated photoacoustic tomography**

ss

Yifan Zhou1‡, Hang Yang1‡, Handi Deng2,3‡, Feng Zhang1, Junjie Jiang1, Sibo Yang1, Xiaodi Sun1, Jiehong Wu1, Yanan Li1, Huijuan Jin1, Hao Wang4, Cheng Ma2,3,5*, Li-Li Li6* and Bo Hu1*

1 Department of Neurology, Union Hospital, Tongji Medical College, Huazhong University of Science and Technology, Wuhan 430022, P. R. China

2 Department of Electronic Engineering, Beijing National Research Center for Information Science and Technology, Tsinghua University, Beijing, 100084, P. R. China

3 Institute for Intelligent Healthcare, Tsinghua University, Beijing, 100084, P. R. China

4CAS Center for Excellence in Nanoscience, CAS Key Laboratory for Biological Effects of Nanomaterials and Nanosafety, National Center for Nanoscience and Technology (NCNST), No. 11 Beiyitiao, Zhongguancun, Beijing, 100190, P. R. China

5 IDG/McGovern Institute for Brain Research at Tsinghua University, Beijing, 100084, P. R. China

6School of Material Science and Engineering Beijing Institute of Technology Beijing 100081, P. R. China

‡These authors contributed equally to this article

*Contact to:

Bo Hu, [hubo@mail.hust.edu.cn](mailto:hubo@mail.hust.edu.cn), Department of Neurology, Union Hospital, Tongji Medical College, Huazhong University of Science and Technology, Wuhan 430022, China;

Li-Li Li, [lill@bit.edu.cn](mailto:lill@bit.edu.cn) , School of Material Science and Engineering Beijing Institute of Technology Beijing 100081, China;

Cheng Ma, [cheng_ma@tsinghua.edu.cn](mailto:cheng_ma@tsinghua.edu.cn), Department of Electronic Engineering, Beijin Nationa Research Center for Information Science and Technology, Tsinghua University, Beijing, 100084, PR China

**Supplementary Experiment Section**

**Materials**

All commercial materials (Macklin, Aladdin, Merck, Sigma-Aldrich, Leyan, Bidepharm) were used without further purification. All solvents were analytical. grade. Products were further characterized using Bruker assisted laser desorption ionization-Time of Flight (MALDI-TOF) mass spectrometry. Analytical TLC was performed on TLC Silica gel 60 F254 25 Aluminium sheets 20x20 cm and flash column chromatography was performed on Qingdao Haiyang Chemical Co. Ltd silica gel 60 (200-300 mesh).

**Synthetic procedures and characterization details**

Final product **9** was prepared according to the synthesized strategy (Fig. S2).

In a 25 mL round-bottom flask, a mixture of acetobromo-α-D-galactose (0.41 g, 1 mmol), 4-hydroxy benzaldehyde (0.12 g, 1 mmol) and silver oxide (0.69 g, 3 mmol) in anhydrous CH_3_CN (5 mL) was stirred overnight at room temperature. The crude mixture used diatomaceous earth to filter. Then the filtrate was extracted with ethyl acetate (3x). The combined organic layers were dried over Na_2_SO_4_, filtered, and concentrated under reduced pressure to give crude product **2** which was used directly without purification.

A solution of NaBH_4_ (0.76 g, 2 mmol ) and product **2** (0.23 g, 0.5 mmol ) in CCl_3_ and IPA（V:V=1:4）5 mL was stirred for 1 h at 0 °C. Then, the solution was warmed to room temperature and stirred for 5 h. After completion of the reaction, the reaction mixture was quenched with water and extracted with EtOAc (3x). Combined organic phase was dried over anhydrous Na_2_SO_4_ and concentrated in vacuo to give crude product **3** which was used directly without purification.

To a solution of **3** (0.23 g, 0.5 mmol) and *para*-nitrophenyl chloroformate (0.20 g, 1 mmol) in CH_2_Cl_2_ (10 mL), was added TEA (0.24 mL, 2 equiv.) at 0 °C. The mixture was stirred for 3 h, hydrolyzed with a saturated solution of NaHCO_3_ and extracted with EtOAc (3x). The combined organic layers were dried over Na_2_SO_4_ and concentrated in vacuo to give crude product **4** which was used directly without purification.

5 mL of DMF was added to a 25 mL round-bottomed flask, the PPa (0.11 g, 0.2 mmol), EDCI (0.77 g, 0.4 mmol), NHS (0.46 mg, 0.4 mmol) were added, and stirred for 8 h at room temperature in darkness condition, then the NH_2_-CH_2_-CH_2_-NH-*t*-BOC (0.05 mL, 1.5 equiv.) was added, and the reaction continued for 12 h after dropping. After completion of the reaction, the reaction mixture was quenched with water and extracted with EtOAc (3x). Combined organic phase was dried over anhydrous Na_2_SO_4_ and concentrated in vacuo to give crude product **6** which was used directly without purification. Then put the crude product in TFA (2 mL) for 4 h. Then use diethyl ether to make the crude product **7** separate out.

To a solution of crude product **4** (0.46 g, 0.75 mmol) and **7** (0.29 g, 0.5 mmol) in DMF (5 mL) was added TEA (0.14 mL, 2 equiv.) at 0 °C. The reaction was stirred at room temperature for 4 h. The combined organic layers were dried over Na_2_SO_4_ and concentrated in vacuo to give crude product **8** which was used directly without purification.

To a solution of crude product **8** (0.11 g, 1 mmol) in MeOH (3 mL) cooled at 0 °C, was dropwise a cold solution (0 °C) of NaOMe (0.48 g, 8.8 mmol) in H_2_O (1 mL). Stirring was continued for 1 h at 0 °C and the solution was neutralized with 1 N HCl adjust the pH to **7**. MeOH was then evaporated and lyophilize the product in a lyophilizer overnight. The crude material was purified by column chromatography over silica gel (CH_2_Cl_2_/MeOH: 20/1, 10/1) to give product **9** as a green solid.

**Microfluidic synthesis of liposomes**

This study employed microfluidic technology to prepare PPA-gal-loaded liposomes. Briefly, DMPC, cholesterol, and DSPE-PEG2000 (molar ratio 60:35:5) were dissolved in anhydrous ethanol to form the lipid phase, while PPA-gal was dissolved in PBS buffer (pH 7.4) to form the aqueous phase. Using a FluidicLab microfluidic device (Shanghai, China), the two phases were rapidly mixed in microchannels at a flow rate ratio (aqueous:organic = 6:1) and a total flow rate of 4 mL/min to form liposomes. The resulting formulation was purified by high-speed centrifugation (15,000×g, 4°C, 30 minutes) to remove unencapsulated drug and organic solvent, with the pellet resuspended in PBS to obtain purified liposomes. Drug loading and encapsulation efficiency were determined by UV-visible spectroscopy: an appropriate amount of liposome dispersion was treated with Triton X-100 (final concentration 0.5%) to disrupt the liposome structure, vortexed, and the absorbance was measured at 710 nm, while simultaneously establishing a standard curve of PPA-gal at different concentrations. The PPA-gal solution before preparation was processed and measured using the same method. Drug concentrations were calculated based on the standard curve, with encapsulation efficiency (EE%) = (amount of encapsulated drug/total amount of drug added) × 100%, and drug loading (DL%) = (amount of encapsulated drug/total weight of liposomes) × 100%.

**In vitro drug release profiles of PPA-gal-LNP**

The in vitro release of PPA-gal-LNP was evaluated using the dialysis method across various physiological conditions. Briefly, 10 mL of PPA-gal-LNP was placed in dialysis bags (MWCO 8 kDa) and immersed in 200 mL of release medium at 37°C with gentle shaking (100 rpm). Three pH environments were tested (pH 7.4, pH 6.8 and pH 5.5 ). At predetermined time points (0-48h), 0.5 mL samples were withdrawn and replaced with fresh medium, and PPA-gal concentration was determined by UV-Vis spectrophotometry.

**NIRF Imaging**

Cy7 dye was encapsulated in liposomes using a standard method and injected into the tail veins of both wild-type and ApoE^−/−^ mice. Six hours later, the mice were euthanized, and their hearts and aortas were imaged. Filters were configured to accommodate Cy7 excitation and emission wavelengths, set at 745 nm and 790 nm respectively. The mean fluorescence intensity of each vessel was quantitatively determined.

**Unmix PA signals**

Due to variations in tissue morphology, the light energy at the aorta differs between individual mice, making direct comparison of the PA signals across different mice inaccurate.1 Therefore, during the imaging process, we used the PA signal of the aorta prior to probe injection as a reference spectrum, denoted as PA₀. The light absorption spectrum of the aorta before probe injection was approximated using the oxyhemoglobin spectrum, denoted as μ₀. We then corrected and transformed the PA spectra at 10 minutes, 2 hours, and 4 hours post-injection, denoted as PAₓ, into light absorption spectra μₓ, using the formula μₓ = (PAₓ × μ₀) / PA₀, where x represents 10 minutes, 2 hours, or 4 hours. To account for the mouse's breathing motion, we selectively used PA signals when the mouse was in a relatively stable state, discarding all data where significant breathing motion was observed. To reduce spectral measurement noise, we applied a polynomial fitting (7 order). Thus, we obtained the light absorption spectra of WT mice and 16-week-old ApoE^-/-^ mice at various time points.

**Anatomical localization of photoacoustic imaging and OCT imaging**

An anatomical landmark-based multimodal imaging registration method was employed to precisely align OCT and photoacoustic imaging data. Specifically, the bifurcation point of the superior mesenteric artery (SMA) from the abdominal aorta served as a common anatomical landmark for both imaging modalities. In photoacoustic tomography, the superior mesenteric artery could be directly visualized due to the intrinsic optical absorption properties of hemoglobin; while in OCT imaging, the exact position of the superior mesenteric artery was marked using a 2 mm metallic vascular clip, which appears as a highly scattering feature in the OCT images.

**Photothermal characteristics**

To evaluate the photothermal properties of nanoparticles (NPs), 150 µL of PPA-galactose-LNP aqueous solutions at varying concentrations (0.6 mM, 0.8 mM, 1 mM, 1.2 mM) and deionized water were each placed into 200 µL centrifuge tubes. These samples were then subjected to continuous irradiation by a 710 nm laser. An infrared camera was employed to capture thermal images and record temperature changes every 30 seconds until the maximum temperature was reached. This procedure enabled the generation of temperature rise curves for NPs at different concentrations. Add 150 µL of PPA-galactose-LNP solution to a 200 µL centrifuge tube and irradiate it with a 710 nm laser. Once the temperature stabilizes at its peak, turn off the laser and allow the solution to cool to room temperature. Repeat this process five times, recording the temperature every 30 seconds with an infrared camera to obtain the heating, cooling, and cycle curves of the NPs.

**Supplementary Table**

[M+H]^+^

The MALDI-TOF mass of spectrum of **7**

| Major criteria |  |
| --- | --- |
| - Active inflammation (monocyte/macrophage and sometimes T-cell infiltration) | 1 point |
| - Thin cap with large lipid core | 1 point |
| - Endothelial denudation with superficial platelet aggregation | 1 point |
| - Fissured plaque | 1 point |
| - Stenosis >90% | 1 point |
| Minor criteria |  |
| - Superficial calcified nodule | 0.5 points |
| - Glistening yellow | 0.5 points |
| - Intraplaque hemorrhage | 0.5 points |
| - Endothelial dysfunction | 0.5 points |
| - Outward (positive) remodeling | 0.5 points |

**Supplementary table 1. Plaque Vulnerability Scoring Criteria.**

**Supplementary Figure**

**
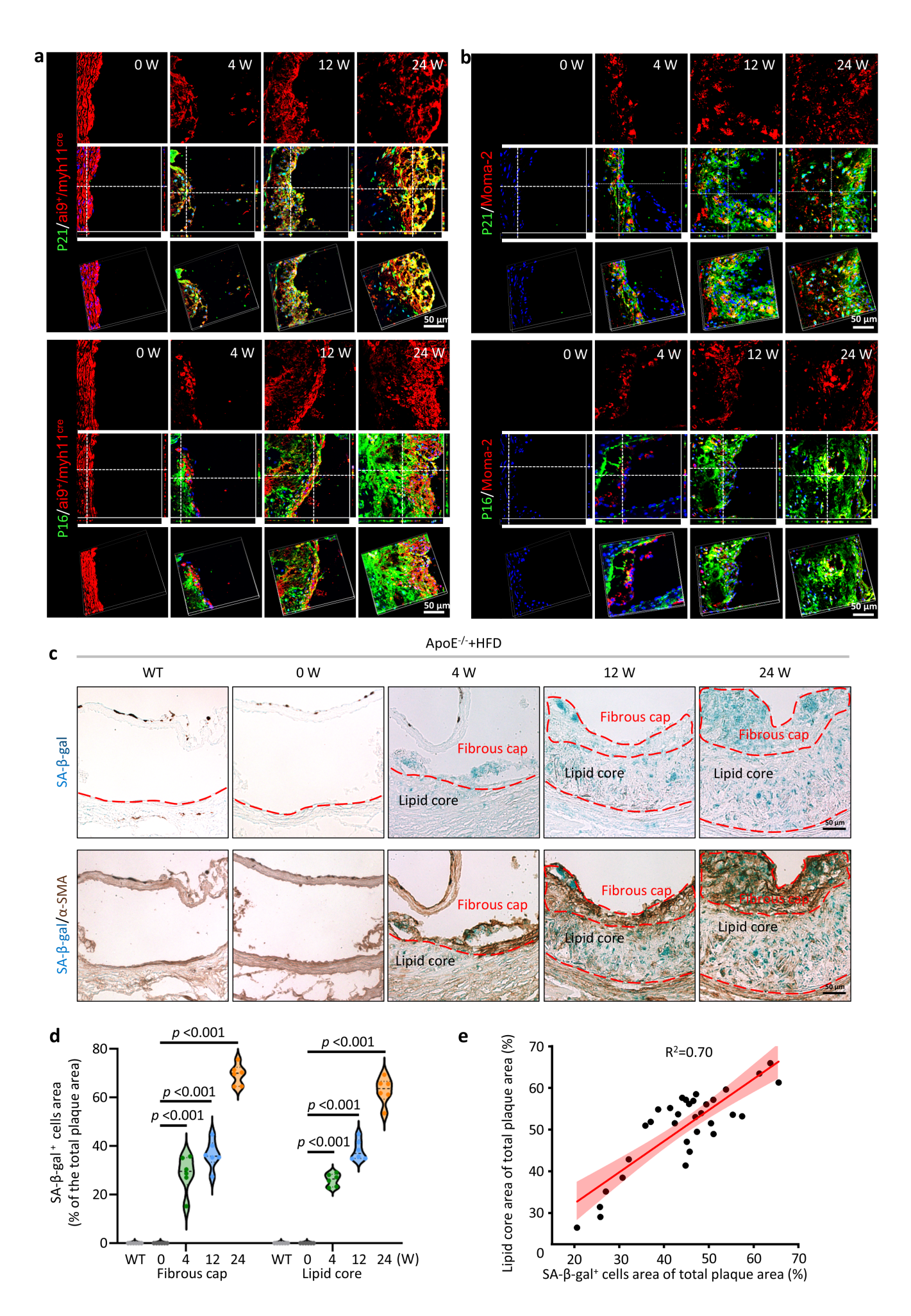
**

**Figure S1. Distribution of senescent cells in atherosclerotic plaques.** a) Co-expression of P16 and P21 (green) with VSMCs (red, ai9) in ApoE^-/-^/ai9^+^/myh11cre^+^ lineage-tracing VSMCs mice. b) Co-expression of P16 and P21 (green) with macrophages (red, MOMA-2). c) Representative images of SA-β-gal stain expressed in mice fed with high fat for different times (week 0, week 4, week 12, week 24). The red dotted line marks the fibrous cap and lipid core of the plaque. d) The proportion of SA-β-gal-stained cells in the fibrous cap and lipid core of plaque tissue at different stages (n = 6). e) Correlation between the proportion of SA-β-gal stained cells and the volume of lipid cores (n=33). Data are represented as mean ± s.d. Statistical significance was assessed by one-way ANOVA with post hoc Tukey’s HSD test (d) and linear regression (e) using GraphPad Prism 9.5. Scale bar = 50 μm.


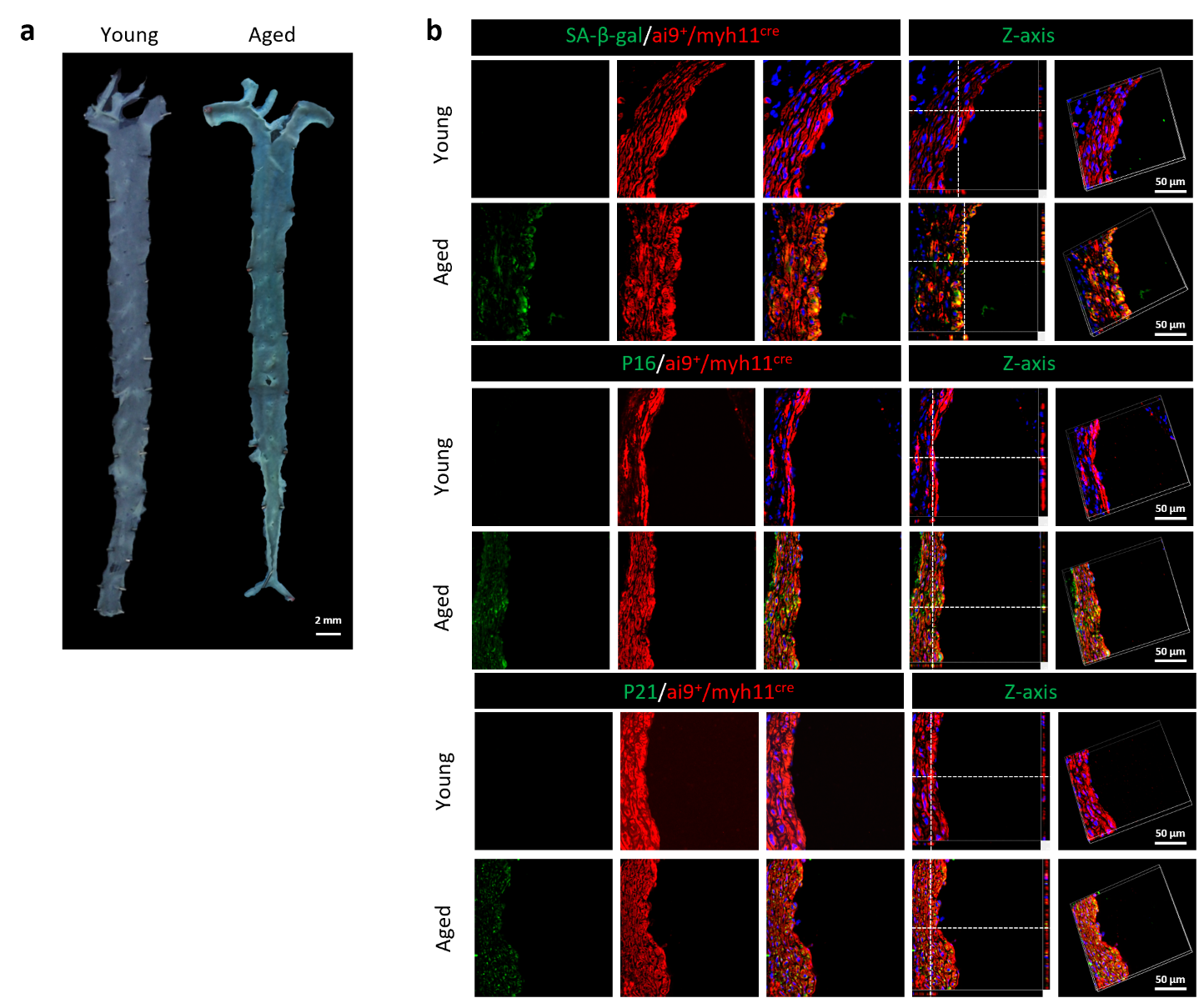


**Figure S2. VSMC senescence in young and aged mice.** (a) Representative SA-β-gal staining of the aorta in young (2-month-old) and aged (24-month-old) mice. (b) Immunofluorescence co-staining reveals the colocalization of senescence markers (SA-β-gal, p16 and p21; green) with lineage-traced VSMCs (ai9; red) in ApoE^-/-^/ai9^+^/myh11cre^+^ lineage-tracing VSMCs mice. Scale bar = 50 μm.


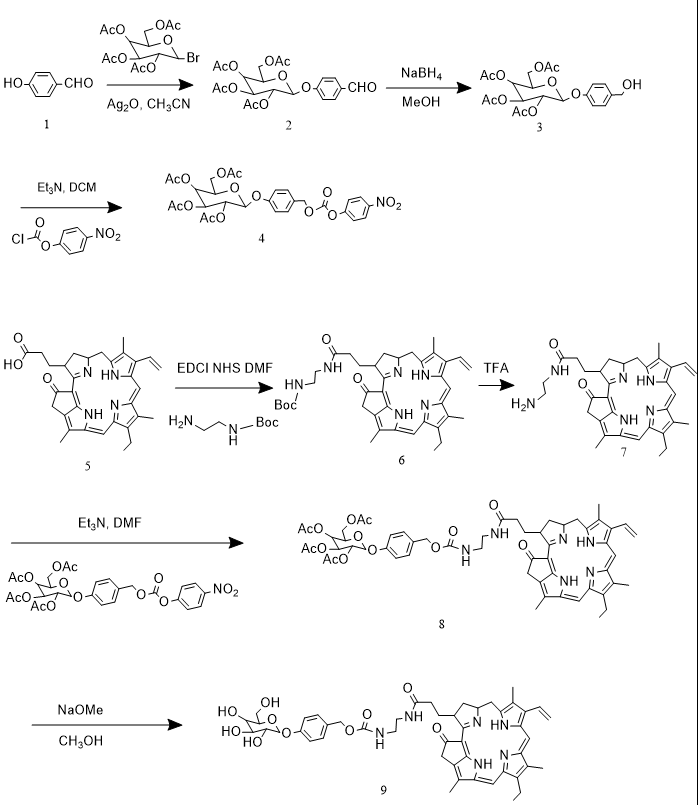


**Figure S3. Schematic synthesized procedure**


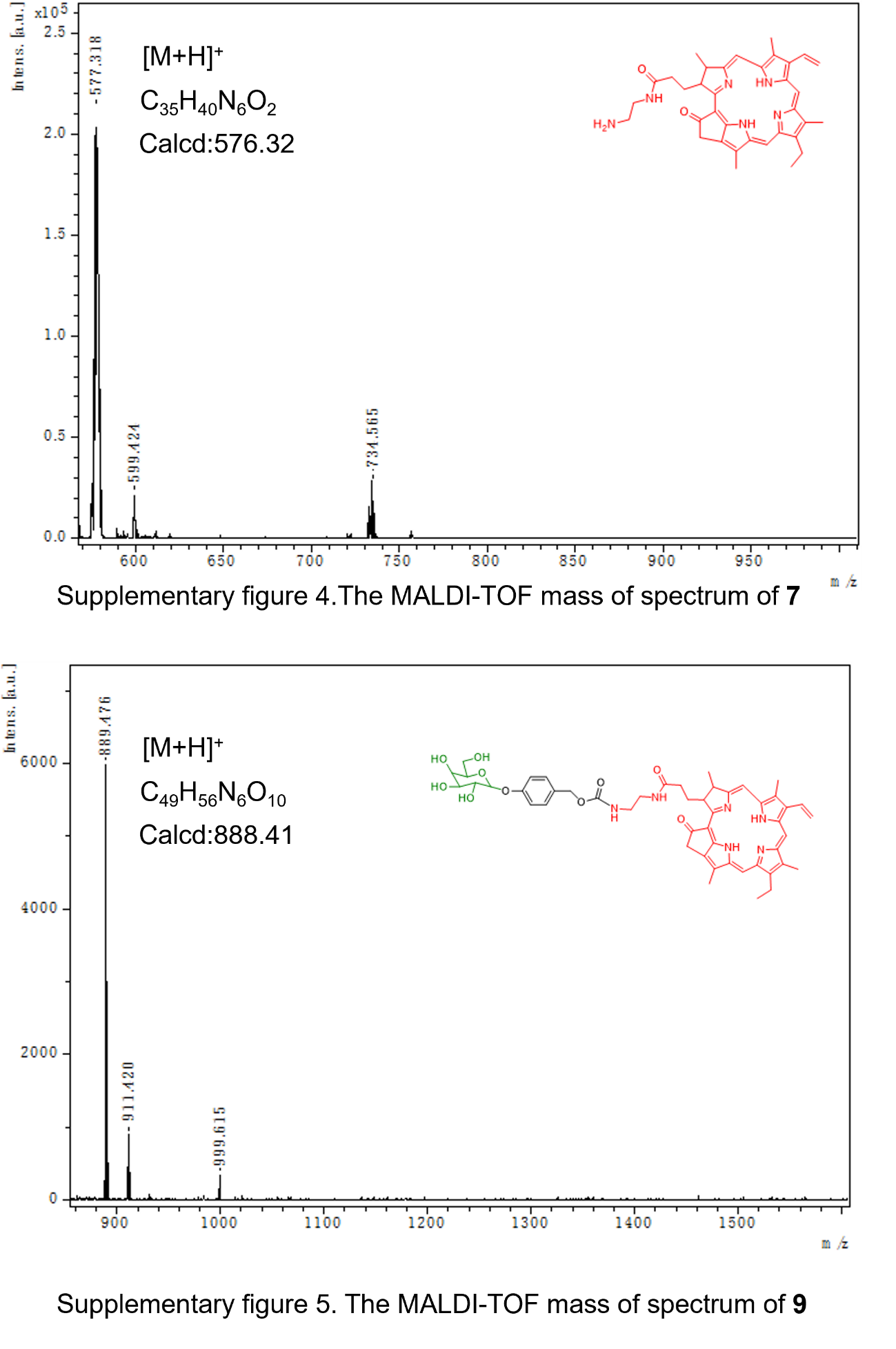


**Figure S4. The MALDI-TOF mass of spectrum of 7**


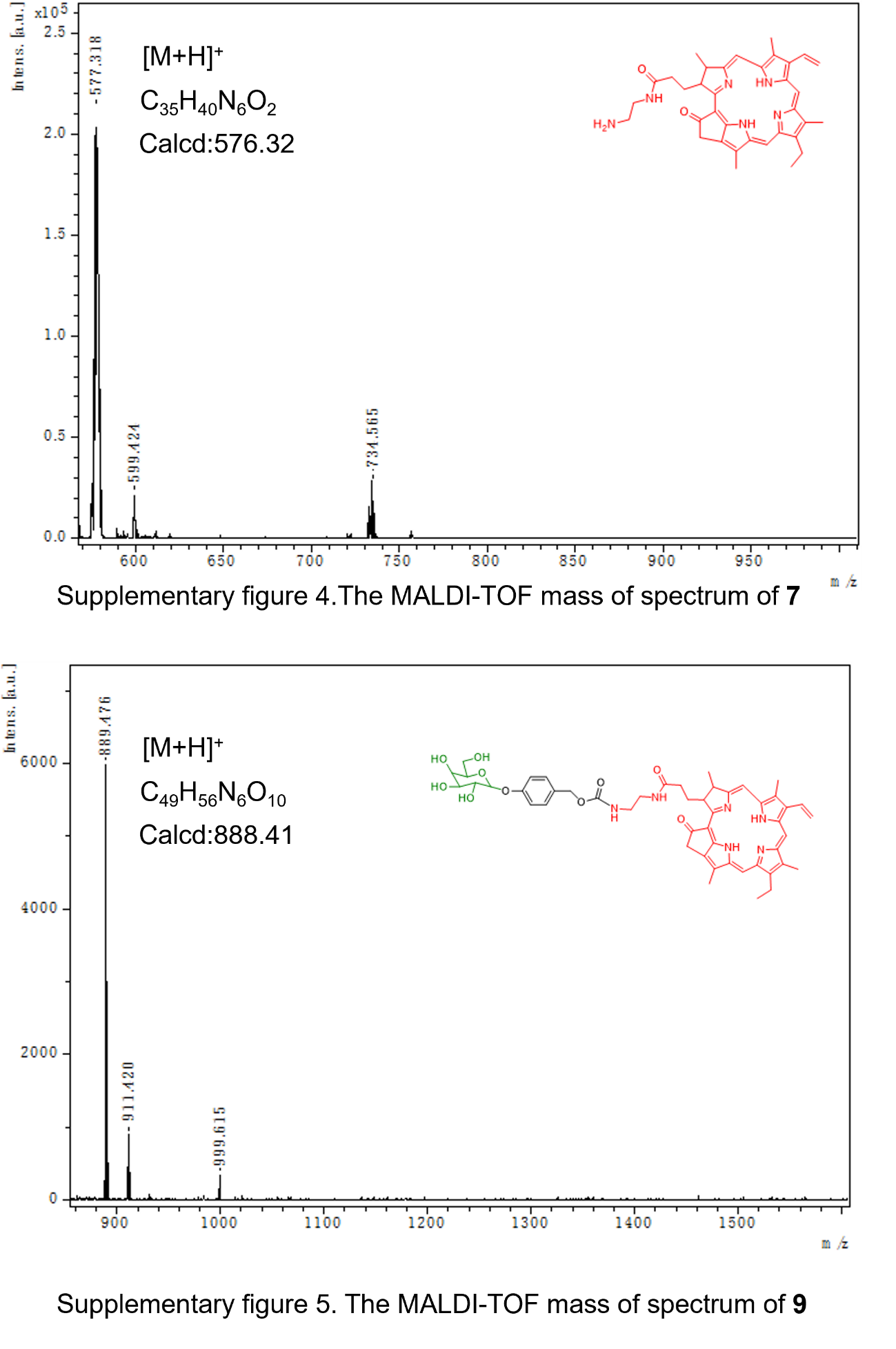


**Figure S5. The MALDI-TOF mass of spectrum of 9**

**
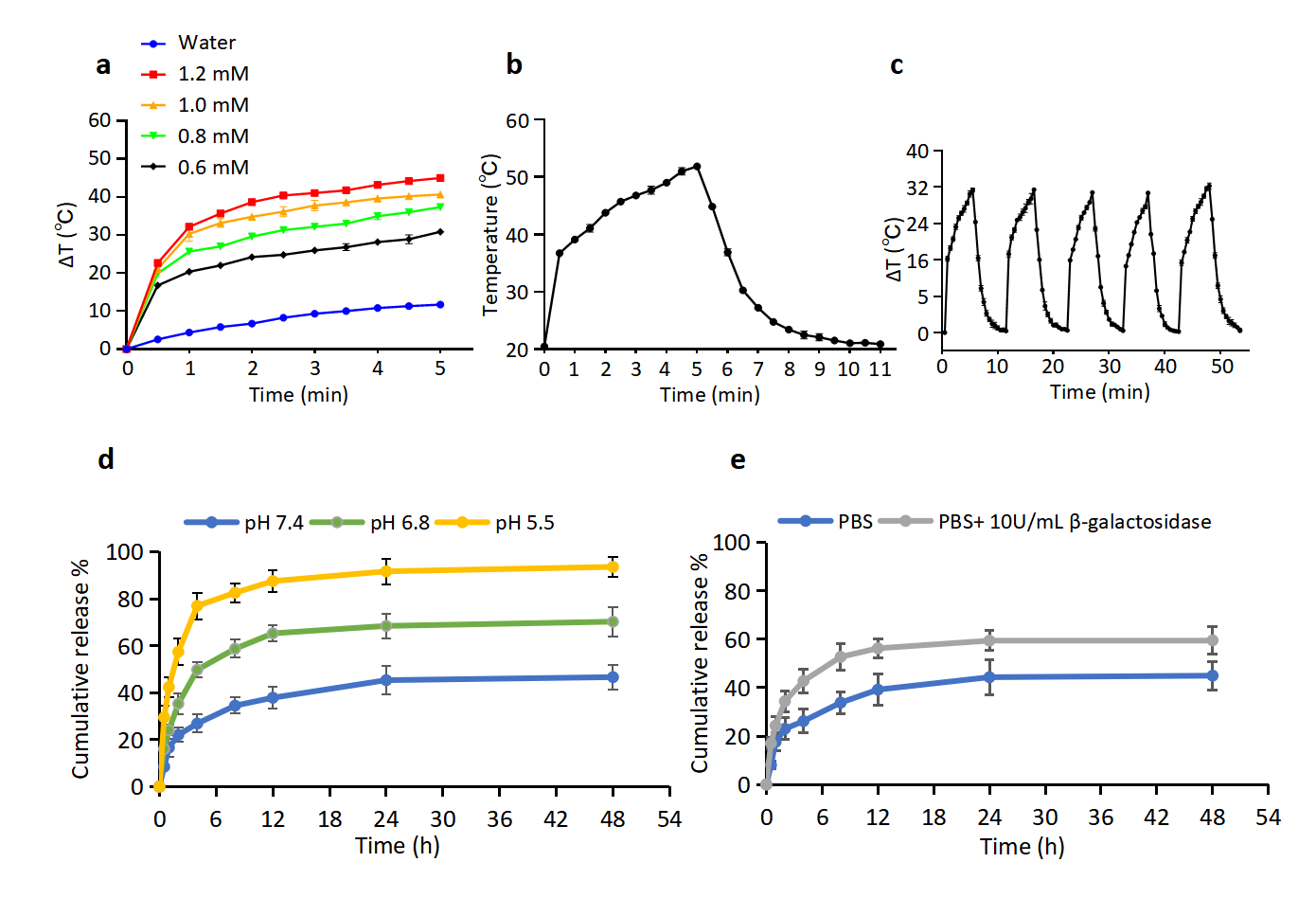
**

**Figure S6. Photothermal properties and release profiles of PPA-galactose-LNP.** a) The photothermal heating curves of PPA-galactose-LNP solution under a 710 nm laser (1 W/cm^2^) treatment (n=6). b) Photothermal effect of an aqueous solution of PPA-galactose-LNP irradiated with 710 nm laser, and the laser was turned off after irradiation for 5 mins (n=6). c) Heating/cooling profiles for four repeated ON–OFF cycles of laser irradiations (n=6). d) Release percentages from PPA-galactose-LNP at pH 7.4, pH 6.8 and pH 5.5(n=6). e) Release percentages from PPA-galactose-LNP in PBS and β-galactosidase solution (10 U/mL) (n=6). Data are represented as mean ± s.d.


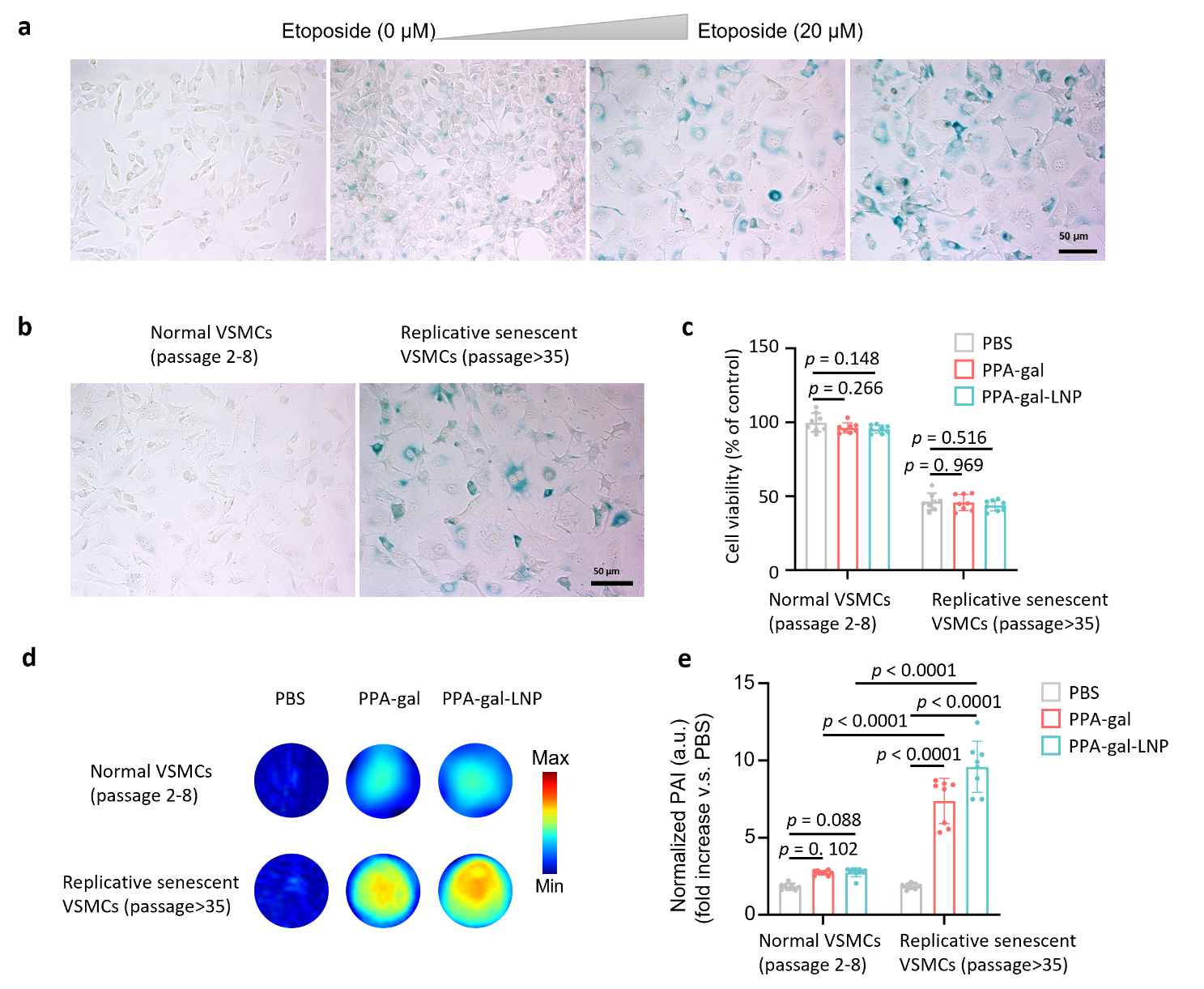


**Figure S7. SA-β-gal staining and photoacoustic imaging of VSMCs.** a) SA-β-gal staining of VSMCs under different concentrations of etoposide treatment. b) Comparative SA-β-gal staining of normal VSMCs and replicative senescent VSMCs. c) Cell viability of normal VSMCs and replicative senescent VSMCs (n=8). d) Representative photoacoustic images of normal VSMCs and replicative senescent VSMCs. e) Quantitative analysis of photoacoustic signal intensity changes in both normal VSMCs and replicative senescent VSMCs (n=8). Scale bar = 50 μm. Data are represented as mean ± s.d. Statistical significance was assessed by one-way ANOVA with post hoc Tukey’s HSD test using GraphPad Prism 9.5.


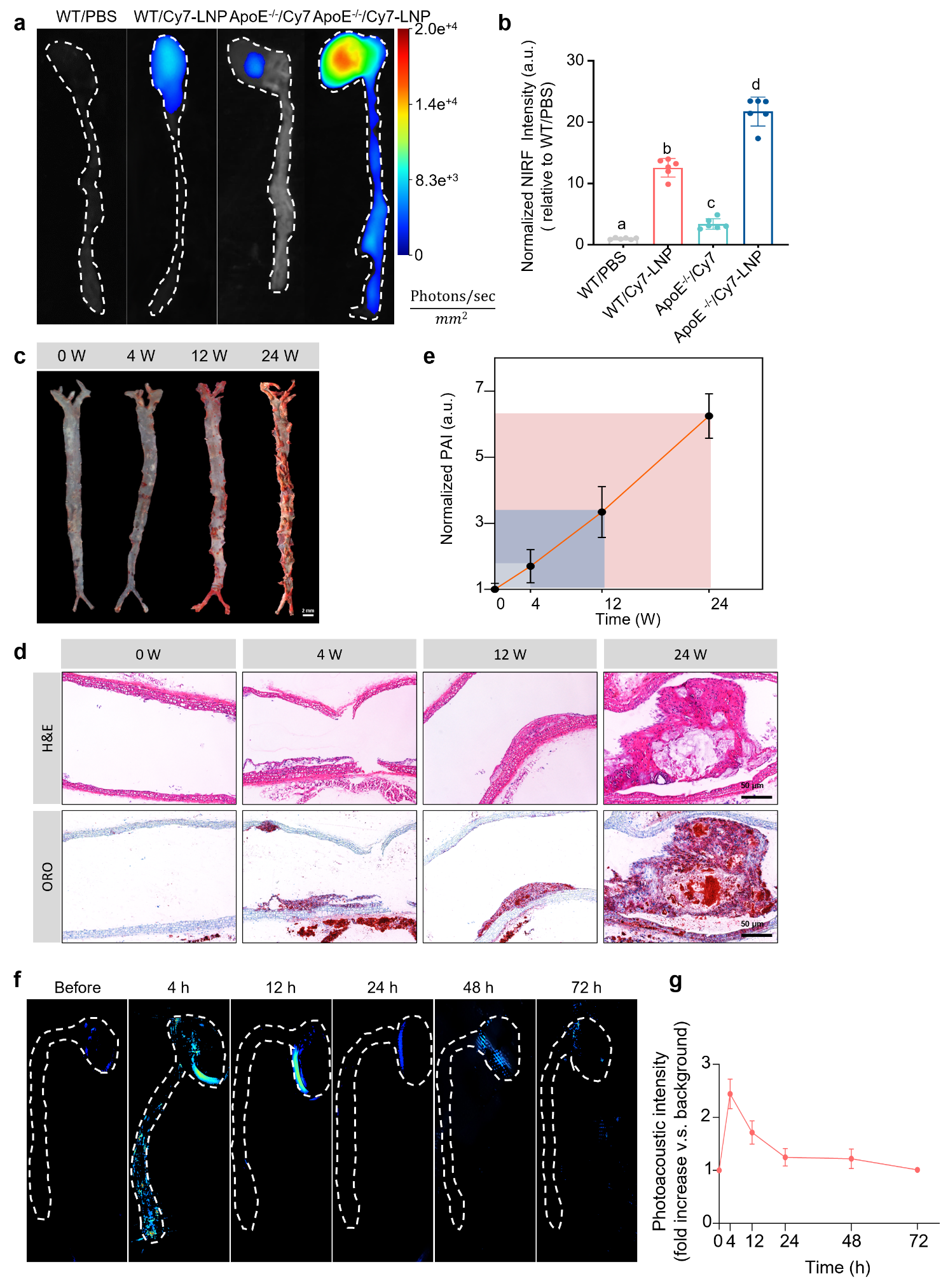


**Figure S8. Characteristics of PPA-galactose-LNP and ORO staining of plaques.** a) Near-infrared imaging of nanoliposome enrichment in the aorta. b) Quantitative analysis of nanoliposome enrichment in the aorta (n=6). Significant differences in all pairwise comparisons of a,b,c,d groups. c) ORO staining of the aorta at different time points during high-fat diet feeding (scale bar = 2 mm). d) ORO and H&E staining of ApoE^-/-^ mice from 0-24 weeks of high-fat diet (scale bar = 50 µm). e) Photoacoustic signal intensities corresponding to different ORO-stained regions (n=6). f) Photoacoustic imaging of nanoliposome enrichment in the aorta at different time points. g) Quantitative analysis of photoacoustic intensity changes of nanoliposomes in the aorta at different time points (n=6). Data are represented as mean ± s.d. Statistical significance was assessed via a one-way ANOVA with post hoc Tukey’s HSD test using GraphPad Prism 9.5.


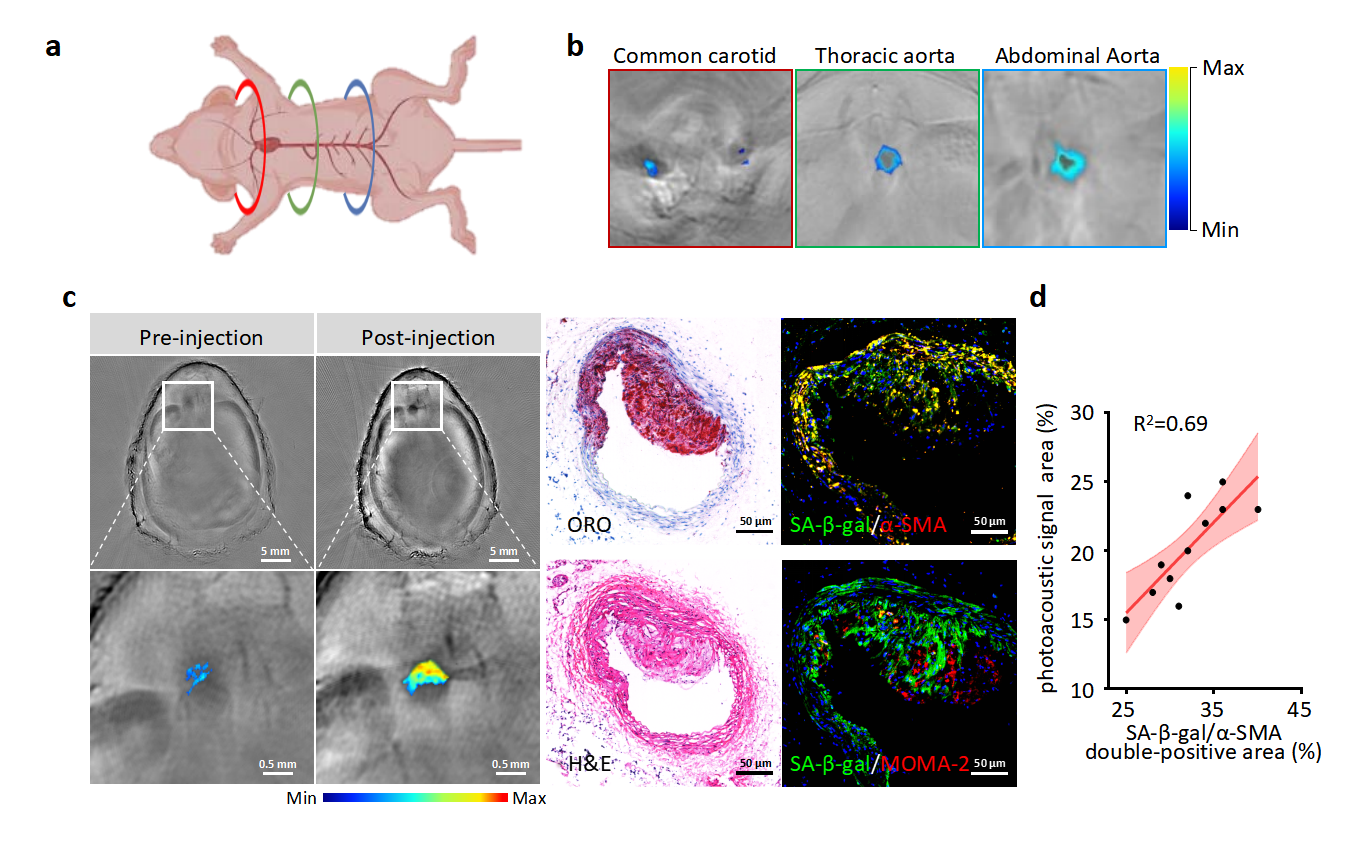


**Figure S9. In vivo photoacoustic imaging of PPA-galactose-LNP.** a) Schematic diagram of the photoacoustic tomography site. b) Cross-sectional scanning of the common carotid artery, thoracic aorta, and abdominal aorta. c) Photoacoustic tomography images (scale bar = 5 mm) and partial zoom-in (scale bar = 0.5 mm) at different time points. H&E and ORO staining of corresponding sections (scale bar = 50 μm), and immunofluorescence of SA-β-gal (green), macrophages (MOMA-2, red), and VSMCs (α-SMA, red) (scale bar = 50 μm). d) Linear correlation between the photoacoustic signal intensities and SA-β-gal^+^ α-SMA^+^ area (n=11).


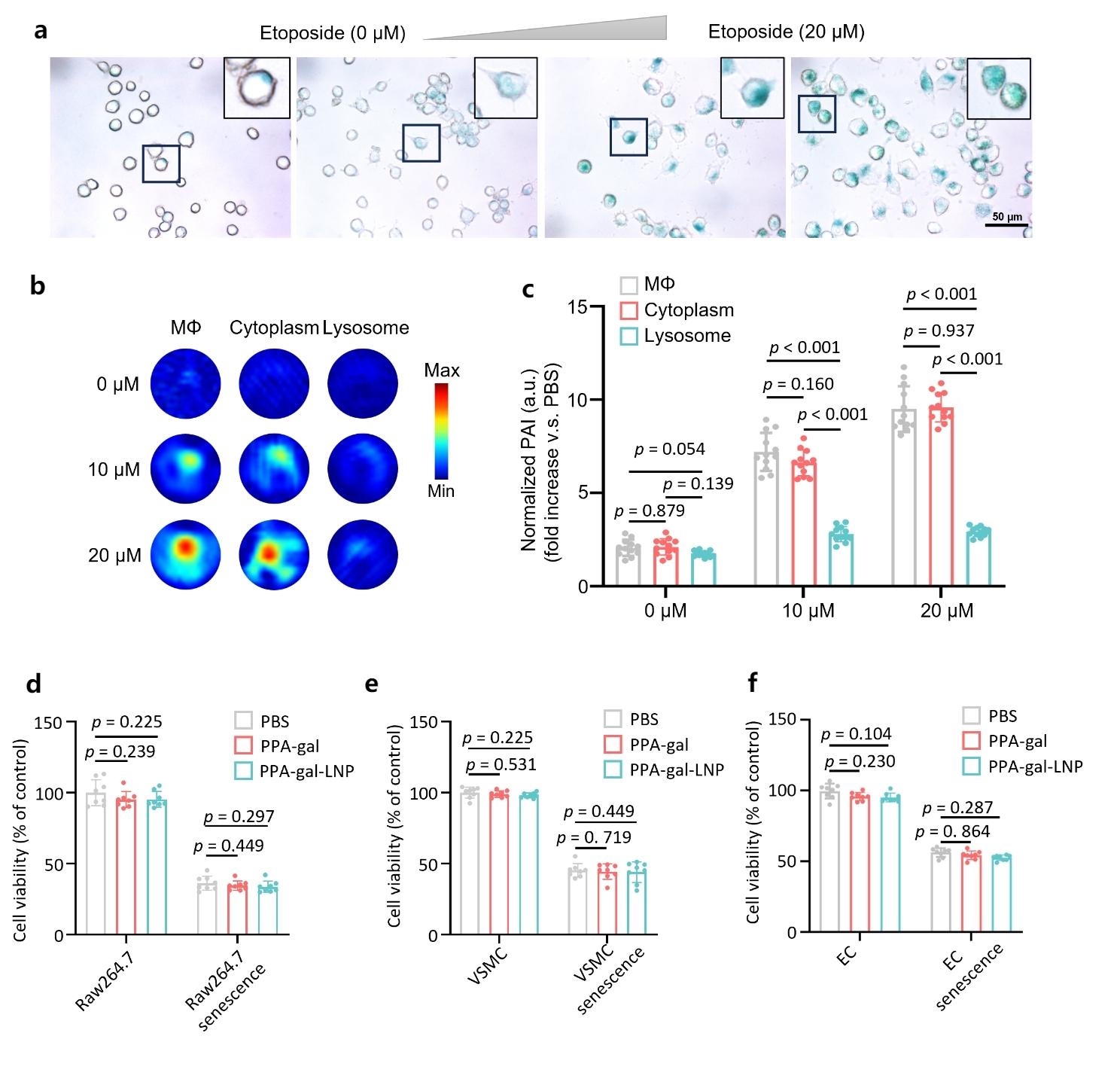


**Figure S10. Lysosomal and cytoplasmic photoacoustic signal detection and viability assessment *in vitro* after PPA-galactose-LNP treatment.** a) SA-β-gal staining in RAW264.7 macrophages treated with different concentrations of etoposide treatment (scale bar = 50 μm). b) Photoacoustic imaging of PPA-gal-LNP distribution in lysosomes and cytoplasm of normal and senescent RAW264.7 cells. c) Quantification of photoacoustic signal intensity differences in lysosomal and cytoplasmic compartments across senescence levels (n=12). (d-f) Cell viability of normal versus senescent VSMCs, RAW264.7 macrophages, and endothelial cells (n=8). Data are represented as mean ± s.d. Statistical significance was assessed via a one-way ANOVA with post hoc Tukey’s HSD test using GraphPad Prism 9.5.


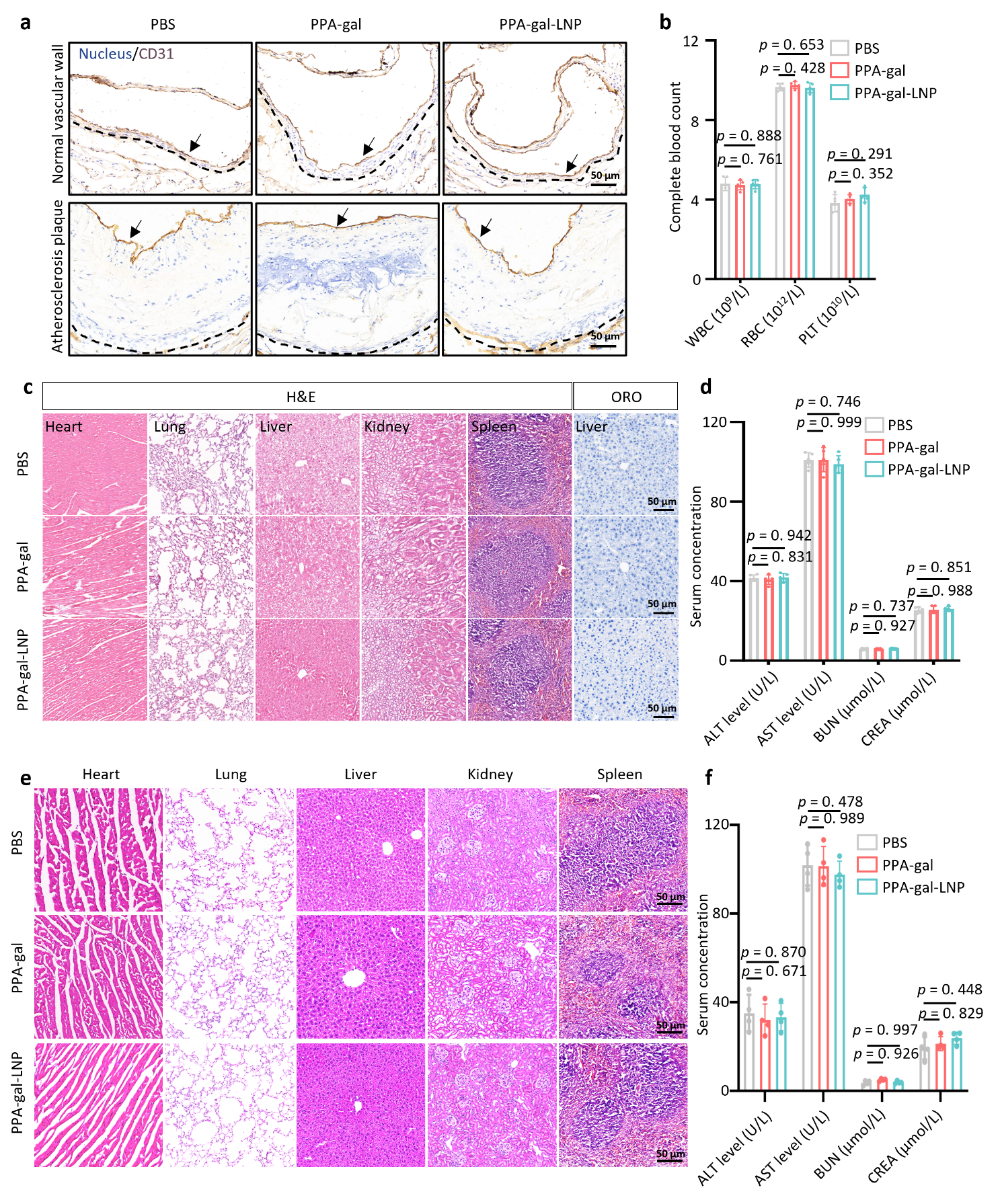


**Figure S11. Biological security of PPA-galactose-LNP.** a) Immunohistochemical staining images of CD31 in normal tissue and atherosclerotic plaques. b) The levels of WBC, RBC, and PLT in the blood of each group were determined by blood biochemical and routine analysis (n = 5). WBC: white blood cells, RBC: red blood cells, PLT: platelets, HGB: hemoglobin. Long-term safety assessment (8 weeks post-injection): (c) Representative H&E-stained sections of major organs (heart, lung, liver, kidney, spleen) and ORO staining of liver tissue following PPA-galactose-LNP administration. d) The levels of AST, ALT, BUN, and CREA at 8 weeks post-treatment (n=5). AST: aspartate transaminase, ALT: alanine transaminase, BUN: blood urea nitrogen, CREA: creatinine. Acute safety assessment (after 3 consecutive daily injections):(e) H&E-stained sections of major organs collected 7 days after repeated PPA-galactose-LNP administration. (f) The levels of AST, ALT, BUN, and CREA at 7 days post-treatment (n=4). Scale bars = 50 μm. Data are represented as mean ± s.d. Statistical significance was assessed via a one-way ANOVA with post hoc Tukey’s HSD test using GraphPad Prism 9.5.

References

[1] Mucong L , Yuqi T , Junjie Y .Photoacoustic tomography of blood oxygenation: A mini review[J]. Photoacoustics, 2018, 10:65-73.DOI:10.1016/j.pacs.2018.05.001.
